# Supplementary material for: Sustained E2F-Dependent Transcription Is a Key Mechanism to Prevent Replication-Stress-Induced DNA Damage
Source: Cell Rep. 2016 May 5;15(7):1412–22. doi: 10.1016/j.celrep.2016.04.036 (PMC4893157; doi:10.1016/j.celrep.2016.04.036)
Supplement: Document S1. Supplemental Experimental Procedures and Figures S1–S5 [file mmc1.pdf]

Cell Reports, Volume *15*

Supplemental Information

Sustained E2F-Dependent Transcription Is a Key Mechanism to Prevent Replication-Stress-Induced DNA Damage

Cosetta Bertoli, Anna E. Herlihy, Betheney R. Pennycook, Janos Kriston-Vizi, and Robertus A.M. de Bruin

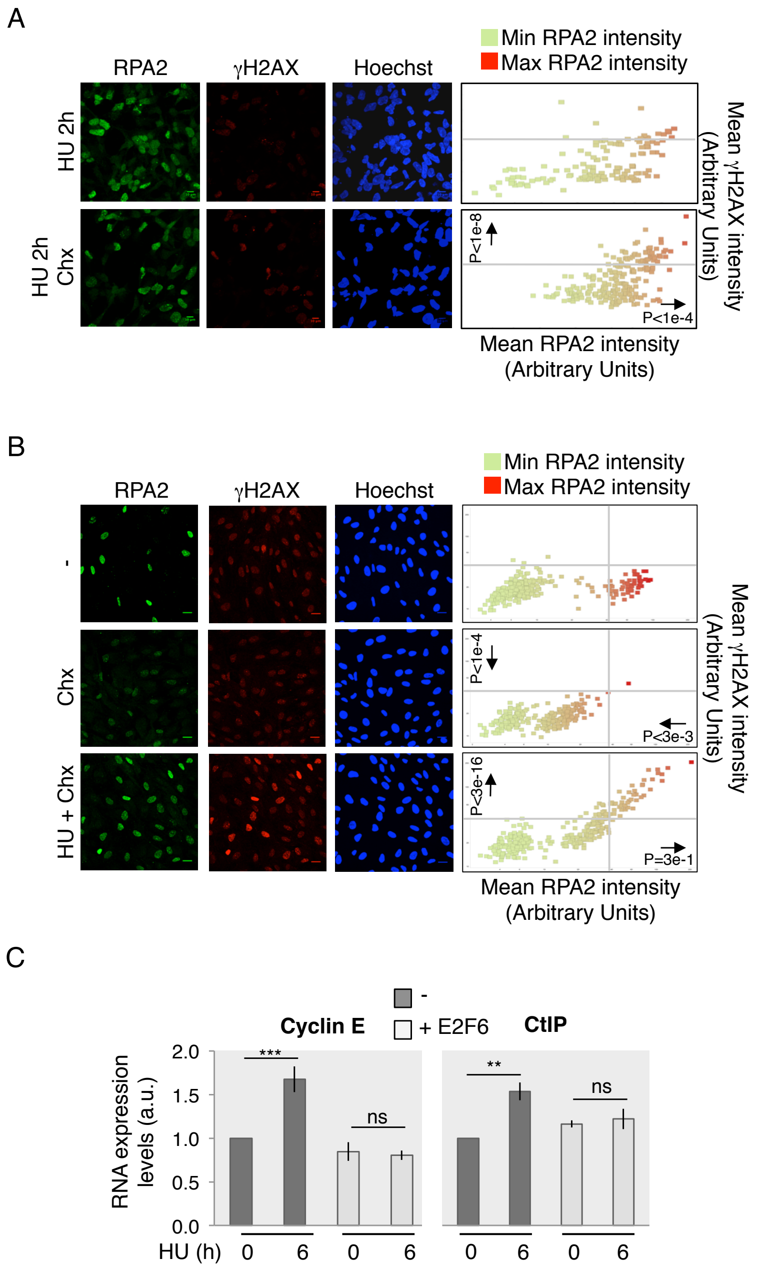

Figure S1, related to Figure 1. Active protein synthesis is required to prevent RS-induced DNA damage. A) Right, scatter plot of mean intensity of RPA2 (green, x-axis) against γH2AX (red, y-axis) of single nuclei, 2 hr HU -/+ Cycloheximide (Chx) in HEK293 T-Rex E2F6 cells. The logarithmic scale is identical for all. P values show significant differences on both axes with the Wilcoxon test. Arrows show increase/decrease of mean on each axis. Left, Representative immunofluorescence images showing chromatin-bound protein. Scale bar represents 10 µm. **B)** Right, scatter plot of mean intensity of RPA2 (green, x-axis) against γH2AX (red, y-axis) of single nuclei. Treatments shown, 7h, RPE1 cells. The logarithmic scale is identical for all. P values show significant differences (except for RPA2 between no treatment and HU + Chx) on both axes with the Wilcoxon test compared to control (-). Arrows show increase/decrease of mean on each axis. Left, Representative immunofluorescence images showing chromatin bound proteins. Scale bar represents 20 µm. **C)** RNA expression of HEK293 T-Rex E2F6 cells after HU treatment -/+ E2F6 overexpression (Doxy 2 µg/ml, 2 hour pretreat) for the time points shown. RNA was extracted and quantified by RT qPCR, normalised to GAPDH and normalised to HU time point 0. n=3, error bars show SEM. *** P<0.001, ** P<0.01 with ANOVA and Tukey's.

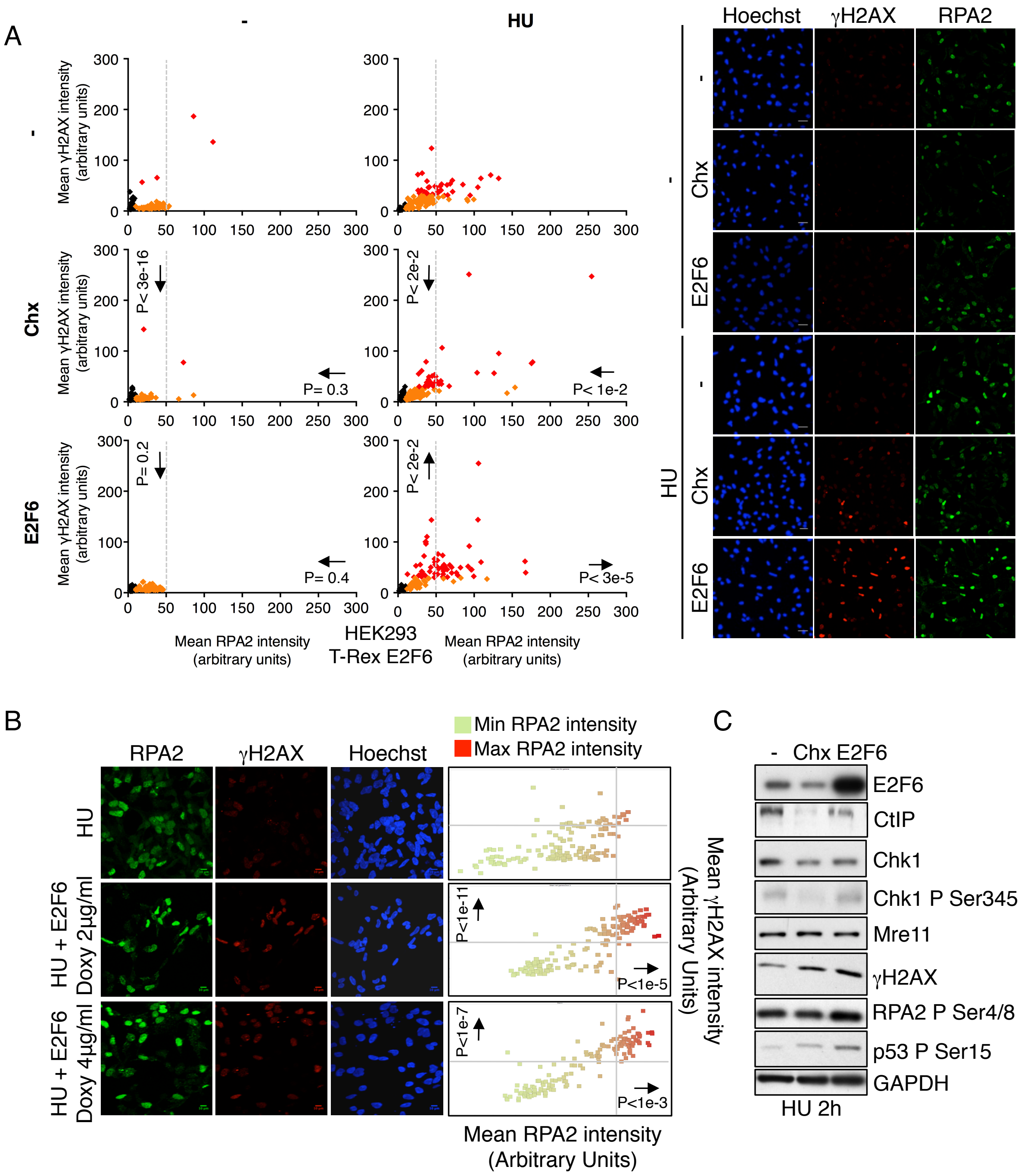

Figure S2, related to Figure 1. Active protein synthesis and E2F transcription is required to prevent RS-induced DNA damage. A) Left, scatter plot of mean intensity of RPA2 (green, x-axis) against γH2AX (red, y-axis) of single nuclei. Treatments shown, 2 hr HU, HEK293 T-Rex E2F6. Black dots show non S phase cells (RPA2<10 a.u.), orange and red dots show low and high levels of γH2AX respectively (arbitrary threshold γH2AX=30 a.u.). P values show differences of S phase cells on both axes with the Wilcoxon test compared to control (- or HU treated as appropriate). Arrows show increase/decrease of mean on each axis. Right, representative images. Scale bar represents 20 µm. **B)** Right, scatter plot of mean intensity of RPA2 (green, x-axis) against γH2AX (red, y-axis) of single nuclei. Treatments shown, 2 hr HU, HEK 293 T-Rex E2F6 cells. Doxy 1.5 hr pretreat. The logarithmic scale is identical for all. P values show significant differences on both axes with the Wilcoxon test compared to control (HU). Arrows show increase/decrease of mean on each axis. Left, Representative immunofluorescence images showing chromatin bound proteins. Scale bar represents 10 µm. **C)** Western blot of WCE, HEK293 T-Rex E2F6 cells, treatments shown, 2h HU. E2F6 (Doxy, 2 µg/ml, 2 hr pretreat).

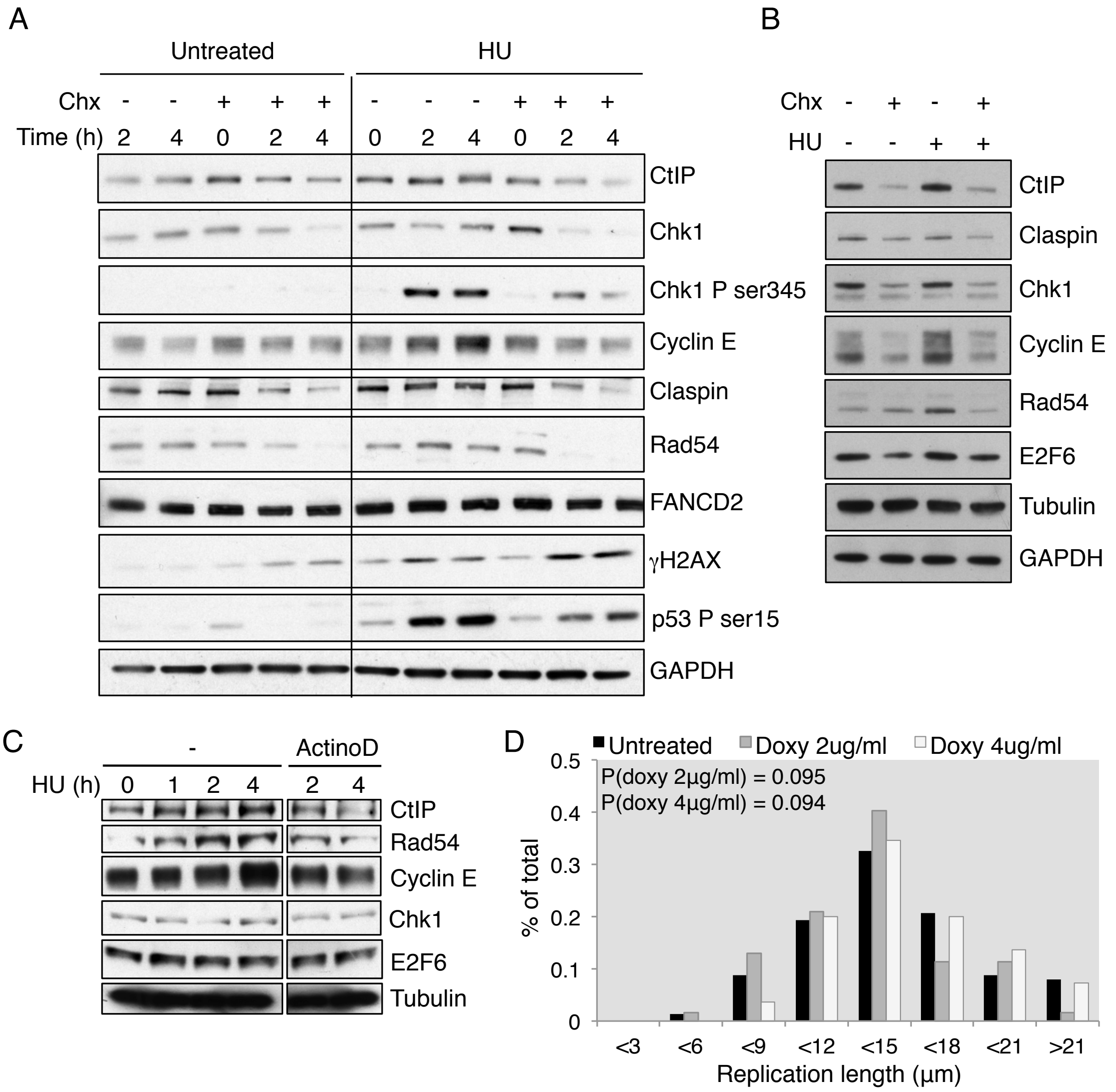

Figure S3, related to Figure 2 and 3. Protein synthesis and E2F transcription are required to maintain the levels of checkpoint proteins during RS. A) Western blot of WCE, T98G cells, treatments and times shown. **B)** Western blot of WCE, RPE1 cells, treatments shown, 4 h. **C)** Western blot of WCE, HEK293 T-Rex E2F6 cells, treatments and times shown. ActinoD is transcriptional inhibitor Actinomycin D. **D)** DNA fiber analysis in HEK293 T-Rex E2F6 cells. Bar graphs of green track lengths. Cells were treated for 3.5 hr -/+ Doxycycline at 2 µg/ml or 4 µg/ml as indicated (timing equivalent to 1.5 hr pretreat then 2 hr HU treatment with Doxy in Figure 3A). Cells were pulse labelled with CldU (red) for 20 min, then IdU (green) for 20 min and the lengths of green tracks measured. P values show no significant difference in track length with E2F6 overexpression with the Student's T test.

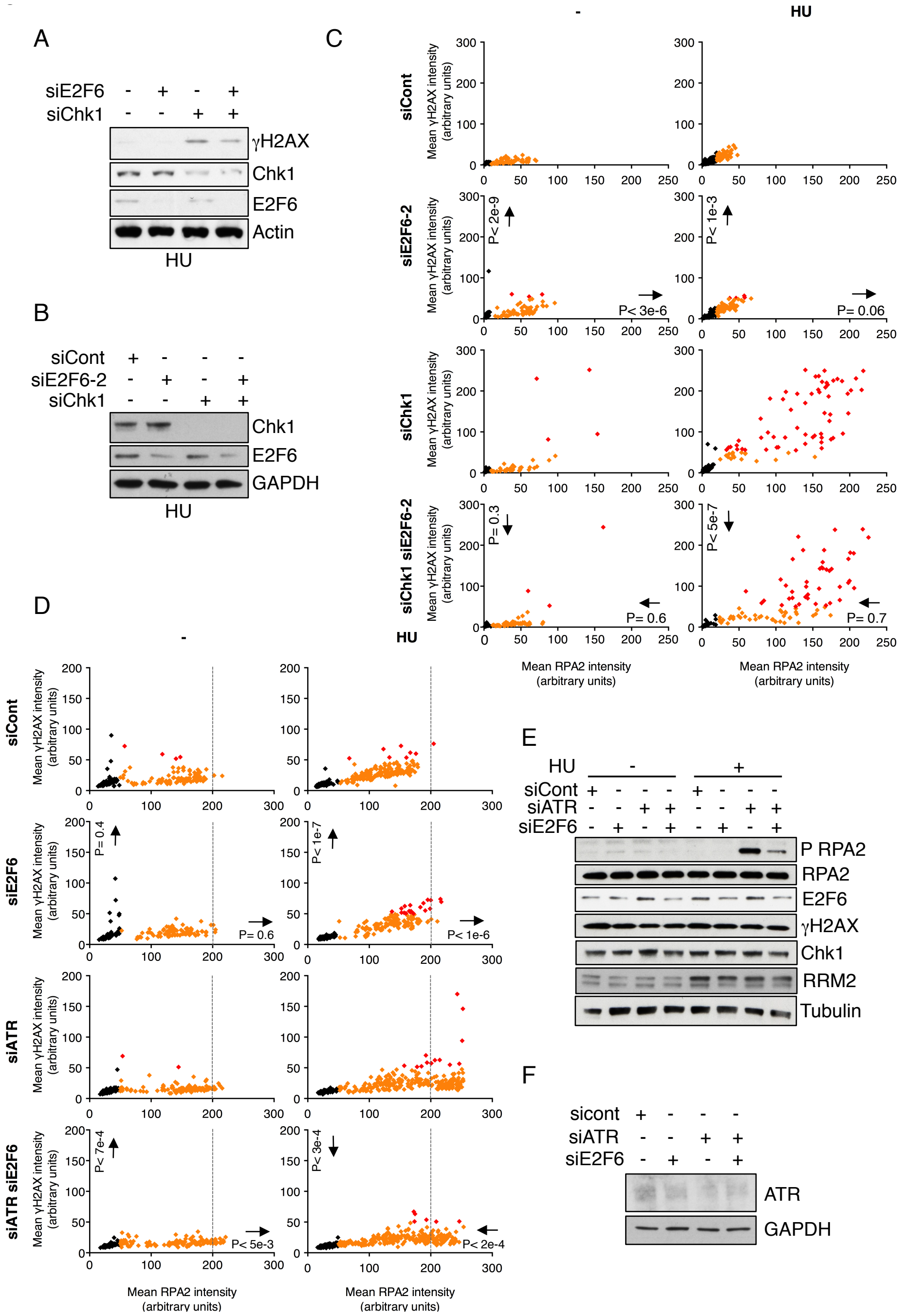

Figure S4, related to Figure 4. E2F activity is required and sufficient to reduce RS-induced DNA damage in a compromised checkpoint response. A) Western blot WCE of serum starvation-synchronized T98G cells transfected with siRNA shown, and released in complete medium with HU for 30 hours. **B)** Western blot of WCE, RPE1 cells, transfected with the reported siRNA, 4h HU. **C)** Scatter plot of mean intensity of RPA2 (x-axis) against γH2AX (y-axis) of single nuclei transfected with the reported siRNA -/+ 4 hr HU, RPE1 cells. Black dots show non S phase cells (RPA2<20 a.u.), orange and red dots show low and high levels of γH2AX respectively (arbitrary threshold γH2AX=50 a.u.). P values show differences on both axes of S phase cells (RPA2 > 20 a.u) with the Wilcoxon test compared to the non-siE2F6 equivalent. Arrows show increase/decrease of mean. **D)** Scatter plot of mean intensity of RPA2 (x-axis) against γH2AX (y-axis) of single nuclei transfected with the reported siRNA, -/+ 4 hr HU, RPE1 cells. Black dots show non S phase cells (RPA2<50 a.u.), orange and red dots show low and high levels of γH2AX respectively (arbitrary threshold γH2AX=50 a.u.). P values show differences on both axes with the Wilcoxon test compared to the non siE2F6 equivalent. Arrows show increase/decrease of mean. **E)** Western blot of WCE, RPE1 cells transfected with the reported siRNA -/+ 4 hr HU. **F)** Western blot of WCE, RPE1 cells transfected with the reported siRNA.

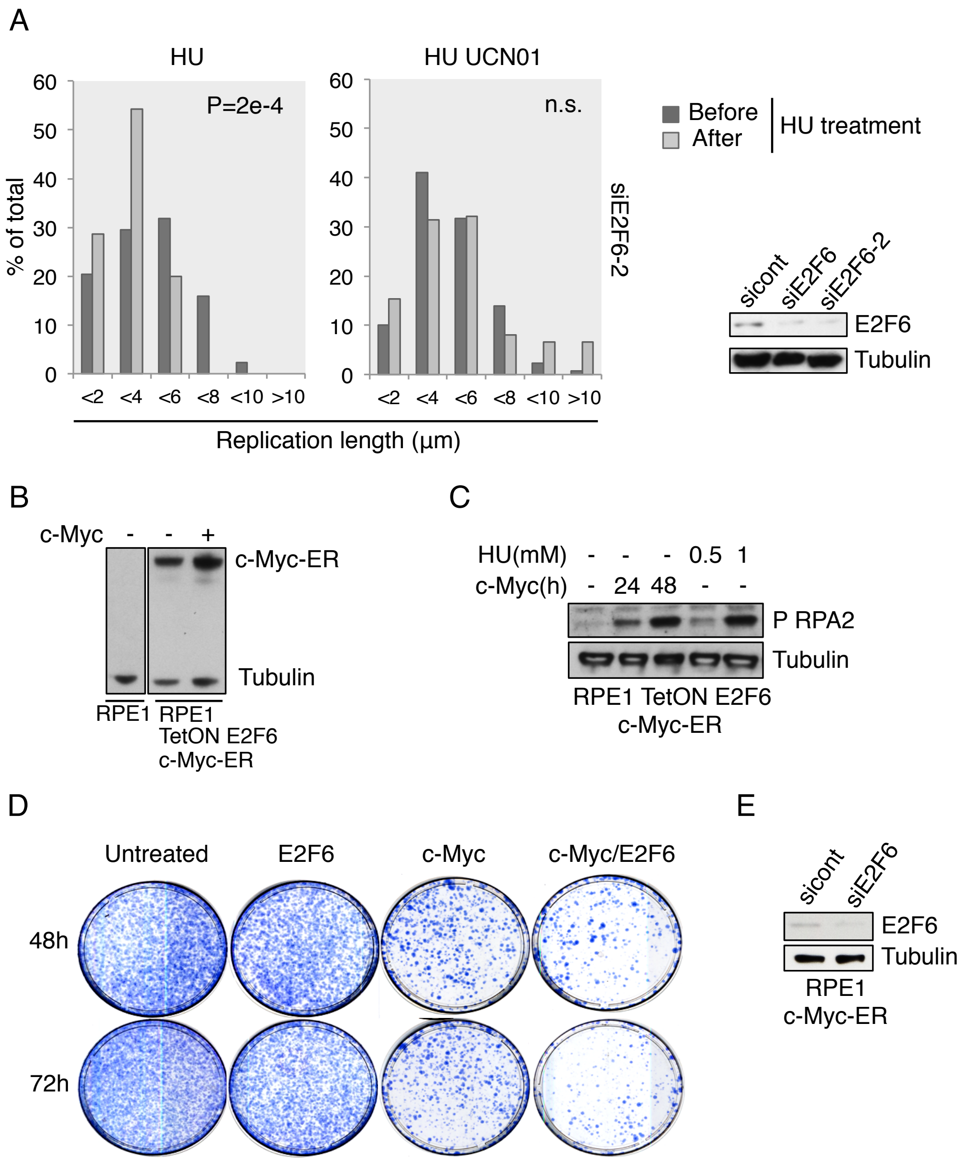

Figure S5, related to Figure 4 and Figure 5. E2F activity is required for tolerance to oncogene-induced RS. **A)** Experimental design as in Figure 4E, T98G cells. Left, bar graphs of length of DNA tracks before and after HU treatment with a second siRNA targeting E2F6 (E2F6-2) -/+ UCN01. P values show difference with the Wilcoxon test. Right, western blot showing the silencing of E2F6 with the two siRNA compared to control siRNA. **B)** Western blot showing the expression of c-Myc-ER in RPE1 TetON E2F6 c-Myc-ER cells compared to non-infected RPE1. Tubulin is the loading control. **C)** Western blot of WCE, RPE1 TetON E2F6 c-Myc-ER cells treated with different RS-causing agents – c-Myc overexpression (times shown) or HU 7 hr (concentrations shown). **D)** Colony formation assays showing the survival and proliferation of RPE1 TetON E2F6 c-Myc-ER cells exposed to c-Myc-ER induction (4OH-T, 100nM), E2F6 overexpression (Doxy 2 µg/ml), or a combination as shown, for the reported time, then diluted in normal medium and allowed to grow for 10 days before being fixed and stained. **E)** Western blot of WCE, RPE1 TetON E2F6 c-Myc-ER treated with the shown siRNA, samples from Figure 5E.

Supplemental Experimental Procedures

Cell culture

HEK293 T-Rex E2F6 and T98G cells were maintained in DMEM (Gibco, 41965) 10% FBS (Sigma, F7524) supplemented with Pennicillin/Streptomycin (Gibco, 15140). RPE-1 hTERT and RPE1 TetON E2F6 were maintained in DMEM or DMEM/F12 (Gibco, 31331) supplemented with Sodium Carbonate (Gibco, 25080) and Pennicillin/Streptomycin, as recommended by ATCC. HEK293 T-Rex E2F6 cells were previously described (Bertoli et al., 2013) and were maintained in 5 μg/ml Blasticidin and 100 μg/ml Zeocin. RPE1 TetON E2F6 were created for this study. Cells were transfected with Lipofectamine 2000 (Invitrogen) following manufacturers instructions using the plasmid pcDNA4/TO E2F6. Transformed cells were selected in 5 μg/ml Blasticidin and 200 μg/ml Zeocin and colonies tested for Doxycycline dependent E2F6 expression. RPE1 TetON E2F6 were maintained in 5 μg/ml Blasticidin and 100 μg/ml Zeocin. RPE1 TetON E2F6 c-Myc-ER was created for this study. Cells were obtained by retroviral infection of RPE1 TetON E2F6 using the pBABE c-Myc-ER plasmid (Addgene plasmid 19128) (Ricci et al., 2004). Infected cells were then selected in puromycin 5 μg/ml and the surviving cells used for the assays. The RPE1 TetON E2F6 c-MycER cells were maintained in phenol-free DMEM/F12 10% charcoal-treated bovine serum with the supplements described above and in 5 μg/ml Blasticidin, 100 μg/ml Zeocin and 2 μg/ml Puromycin.

siRNA Transfection

The sequences used were: siCont (LacZ) AACGUACGCGGAAUACUUCGA, siE2F6 AAGGAUUGUGCUCAGCAGCUG (**Figures 4ABCDF, 5E, S4ADE, S5E**), siE2F6-2 AAACAAGGUUGCAACGAAAUU (**Figures S4B, S4C, S5A**), siChk1 GAAGCAGUCGCAGUGAAGAUU (Bertoli et al., 2013) (**Figures 4D, S4A**), SMARTpool: siGENOME CHEK1 siRNA (M-003255-04-005, Dharmacon GE Life Sciences) (**Figures 4ABC, S4B,S4C**), siATR UUGUAGAAAUGGAUACUGA (validated Silencer Select s536, Thermo Fisher Scientific).

Immunofluorescence

Figures 1A, B, 4B, C, 5D, E, and Figures S1A, B, S2A, B, S4C,D : Cells were pre-extracted for 1 min in ice cold PBS 0.2% Triton-X100 (or 0.15% for HEK293 T-Rex cells), then fixed with 4% formaldehyde for 20 min and processed similarly to (Toledo et al., 2013). Briefly, coverslips were blocked in 1% BSA for 1 hr and incubated in primary antibodies overnight at 4˚C – RPA32 (RPA2) (1:500, Ms, MABE285, Millipore) and Phospho-Histone H2A.X (γH2AX) (Ser139) (1:400, Rb, 20E3, Cell Signaling Technology). Coverslips were incubated in secondary antibodies for 1 hour at RT – anti-mouse Alexa Fluor 488, and anti-rabbit Alexa Fluor 647 both 1:2000 (LifeTechnologies), incubated 5 minutes in Hoechst 1:10,000, (Invitrogen), and mounted with Fluoroshield (Sigma). Images were obtained with a Leica TCS SP5, or a Leica TCS SPE2, 63x objective lens. Images were processed in Fiji – the Hoechst channel was used to segment nuclei with a median filter and Otsu thresholding. The mean gray value of each channel was then measured for each nucleus. The signals for individual cells were then plotted using Spotfire or Prism software.

Figure 3C,D: Cells were fixed with 4% formaldehyde for 20 min and permeabilised for 5 min in PBS 0.2% Triton-X100. Coverslips were then processed as above with RPA32 (RPA2) antibody (1:1000, Ms, MABE285, Millipore). The percentage of cells containing RPA2 foci was manually counted in Fiji.

Statistics

Figure 1A,B, 5D,E and Figure S2A, S4C: Statistical significance was analyzed using the Wilcoxon sum rank test; two sided, using the R function Wilcox.test. Only S phase cells were analyzed, defined as the portion of cells where RPA2 > 10 a.u. (Figure 1B, 5E, S2A), RPA2 > 20 a.u. (Figure 5D, S4C), or where RS occurs, as seen by an increase in γH2AX intensity during HU treatment (Figure 1A). **Figures 4B and Figures S1A,B, S2B, S4D:** Statistical significance was analyzed for both RPA2 and γH2AX against control conditions using the Wilcoxon sum rank test, two sided, using the R function Wilcox.test. **Figures 1C, 3A, B and Figure S3D:** Statistical significance was analyzed by Student's T test. **Figure 3C:** Statistical significance was analyzed by two-way ANOVA (using the aov() function of R), the multiple comparisons of the means was performed by Tukey's honest significant differences, using the R function TukeyHSD().*** $P<0.001$, * $P<0.05$. **Figure 4F and Figure**

S5A: Statistical significance was analyzed for differences between mean track length after – mean track length before HU treatment using the Wilcoxon sum rank test, two sided, using the R function Wilcox.test.
Figure S1C: Statistical significance was analyzed by two-way ANOVA, the multiple comparisons of the means was performed by Tukey's honest significant differences, using Prism software. *** P<0.001, ** P<0.01.

Western blot

Cell extracts were prepared in RIPA buffer (Tris-HCl pH7.5 20mM, NaCl 150mM, EDTA 1mM, EGTA 1mM, NP40 1%, NaDoc 1%) containing phosphatase inhibitor cocktails 2 and 3, 1:1000 (Sigma P5726 and P0044) and protease inhibitor cocktail, 1:1000 (Sigma, P8340). Run on Novex 4-12% Bis-Tris protein gels. The following antibodies were used for Western blot analysis, all were used at 1:1000 unless otherwise stated: Claspin (Rb, ab3720), CtIP (Rb, ab70163), Mre11 (Ms, ab214), Rad54 (Ms, ab11055), Histone H3 (Rb, ab1791) from Abcam, Chk1 (Ms, DCS-310, sc-56291), Cyclin E (HE12) (Ms, sc-247), Rad51 (Rb, H-92, sc-8349), Cdc7 (1:200, Ms, DCS-341, sc-56274), PCNA (1:2000, Ms, F-2, sc-25280), ATR (Gt, N-19, sc-1887), Myc 9E10 (sc-40) from Santa Cruz, GAPDH (Ms, GT239) from GeneTex, Tubulin (1:2000, Ms, MAB3408), RPA34-20 (Ms, MABE285), E2F6 (Ms, MABE57) from Millipore, FANCD2 (1:2000, Rb, NB100-182) from Novus biological, RPA32 (RPA2) (S4/S8) (Rb, A300-245A-3) from Bethyl laboratories, Phospho-Histone H2A.X (γH2AX) (Ser139) (Rb, 20E3), Phospho-Chk1 (ser345) (Rb, 2341), p53 (Ser15) (Rb) from Cell Signaling Technology, Actin (Rb, A2066) from Sigma, RRM2 (Ms, M01, 1E1), E2F6 (Ms) a kind gift of Dr Jacky Lees. Secondary antibodies used were Goat anti-mouse and anti-rabbit HRP conjugated (Fisher Scientific, PA1-74421 Mouse, PI-31460 Rabbit).

Fiber analysis

HEK-293 T-Rex E2F6 or T98G were labeled with 25 µM CldU followed by 250 µM IdU, as in the schematics. Fiber spreading and labeling was performed as in (Petermann et al., 2010). Images were taken by confocal microscopy and analyzed with ImageJ. 150-200 fibers were measured for each experiment.

RNA expression

RNA was extracted with the Qiagen RNeasy Mini kit following manufacturers protocol. RT qPCR SYBR assay was carried out with Mesa Blue following manufacturers instructions, 80 ng RNA per well, experimental triplicates, BioRad CTX Connect qPCR machine. RNA levels were normalized to GAPDH. Experiment was done in biological triplicate. Primers used were: GAPDH Fw 5'- GAAATCCCATCACCATCTTCCAGG-3', Rv 5'-GAGCCCCAGCCTTCTCCATG-3', Cyclin E Fw 5'-TGTCCTGGATGTTGACTGCCTTGA-3', Rv 5'-TGTCGCACCACTGATACCCTGAAA-3', CtIP, Fw 5'- CTGCTTGGGCACACGTGTAAG-3', Rv 5'-TGGAATGTAGCGGAATCGGTGTCT-3'.

Chromatin Preparation

Buffer A (Hepes pH 7.9 10 mM; KCl 10 mM; MgCl 1.5 mM; Sucrose 0.34 M; Glycerol 10 %; DTT 1 mM; and protease and phosphatase inhibitors as recommended by the supplier). Buffer B (3 mM EDTA; 0.2 mM EGTA; protease and phosphatase inhibitors). The nuclear fraction was pelleted at 1300 g, 5 min, 4˚C. The chromatin fraction was pelleted at 1700 g, 5 min, 4˚C. The sample was spun at 9600 g, 5 min, 4˚C before use.

FACS

Flow cytometry was performed as described previously for DAPI/RPA2/γH2AX in (Forment et al., 2012). Phospho-Histone H2A.X (γH2AX) (Ser139) (Rb, 20E3) from Cell Signaling Technology used 1:200 and RPA2 (Ms, 9H8, ab2175) from Abcam used 1:250. Samples were measured on a BD LSRII flow cytometer using DIVA software (BD) and analyzed using FlowJo software.

Survival Assay

RPE1 TetON E2F6 c-MycER were treated as shown in Figure S5D for 48 or 72 hours, they were then diluted and left to grow in 10cm dishes until colonies were visible. Cells were fixed and stained as in Bertoli et al, 2013.

Supplemental References

Bertoli, C., Klier, S., McGowan, C., Wittenberg, C., and de Bruin, R.A. (2013). Chk1 Inhibits E2F6 Repressor Function in Response to Replication Stress to Maintain Cell-Cycle Transcription. Curr Biol.

Forment, J.V., Walker, R.V., and Jackson, S.P. (2012). A high-throughput, flow cytometry-based method to quantify DNA-end resection in mammalian cells. Cytometry Part A : the journal of the International Society for Analytical Cytology *81*, 922-928.

Petermann, E., Orta, M.L., Issaeva, N., Schultz, N., and Helleday, T. (2010). Hydroxyurea-stalled replication forks become progressively inactivated and require two different RAD51-mediated pathways for restart and repair. Mol Cell *37*, 492-502.

Ricci, M.S., Jin, Z., Dews, M., Yu, D., Thomas-Tikhonenko, A., Dicker, D.T., and El-Deiry, W.S. (2004). Direct repression of FLIP expression by c-myc is a major determinant of TRAIL sensitivity. Mol Cell Biol *24*, 8541-8555.

Toledo, L.I., Altmeyer, M., Rask, M.B., Lukas, C., Larsen, D.H., Povlsen, L.K., Bekker-Jensen, S., Mailand, N., Bartek, J., and Lukas, J. (2013). ATR prohibits replication catastrophe by preventing global exhaustion of RPA. Cell *155*, 1088-1103.
