# Supplementary figures and images for: Sustained E2F-Dependent Transcription Is a Key Mechanism to Prevent Replication-Stress-Induced DNA Damage
Source: Cell Rep. 2016 May 5;15(7):1412–22. doi: 10.1016/j.celrep.2016.04.036 (PMC4893157; doi:10.1016/j.celrep.2016.04.036)

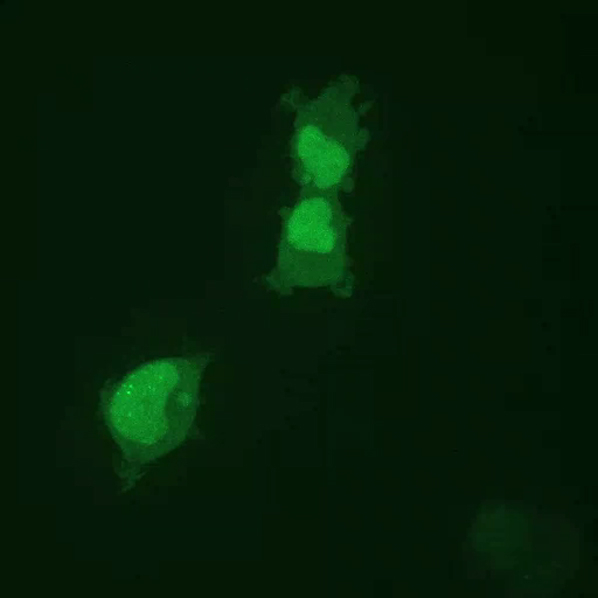

Supplement: Movie S1. RPA2-GFP Foci Resolve when Released from HU Treatment, Related to Figure 3 — Movie of HEK293 T-Rex E2F6 cells stably expressing RPA2-GFP treated for 20 hours with HU, then washed and released into normal medium for up to 20 hours. The imaging was performed with a spinning disk live microscope and images were taken every 20 minutes. [file mmc2.jpg]

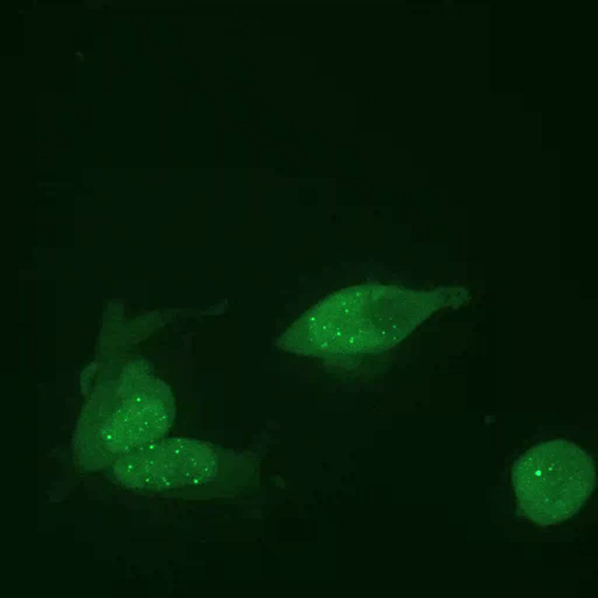

Supplement: Movie S2. RPA2-GFP Foci Resolution Is Impaired when E2F6 Is Overexpressed during HU Treatment, Related to Figure 3 — Movie of HEK293 T-Rex E2F6 cells stably expressing RPA2-GFP treated for 20 hours with HU and E2F6 overexpression (Doxy, 2μg/ml), then washed and released into normal medium for up to 20 hours. The imaging was performed with a spinning disk live microscope and images were taken every 20 minutes. [file mmc3.jpg]
