# Supplementary material for: Sustained E2F-Dependent Transcription Is a Key Mechanism to Prevent Replication-Stress-Induced DNA Damage
Source: Cell Rep. 2016 May 5;15(7):1412–22. doi: 10.1016/j.celrep.2016.04.036 (PMC4893157; doi:10.1016/j.celrep.2016.04.036)
Supplement: Document S2. Article plus Supplemental Information [file mmc4.pdf]

Cell Reports

Report

Sustained E2F-Dependent Transcription Is a Key Mechanism to Prevent Replication-Stress-Induced DNA Damage

Graphical Abstract

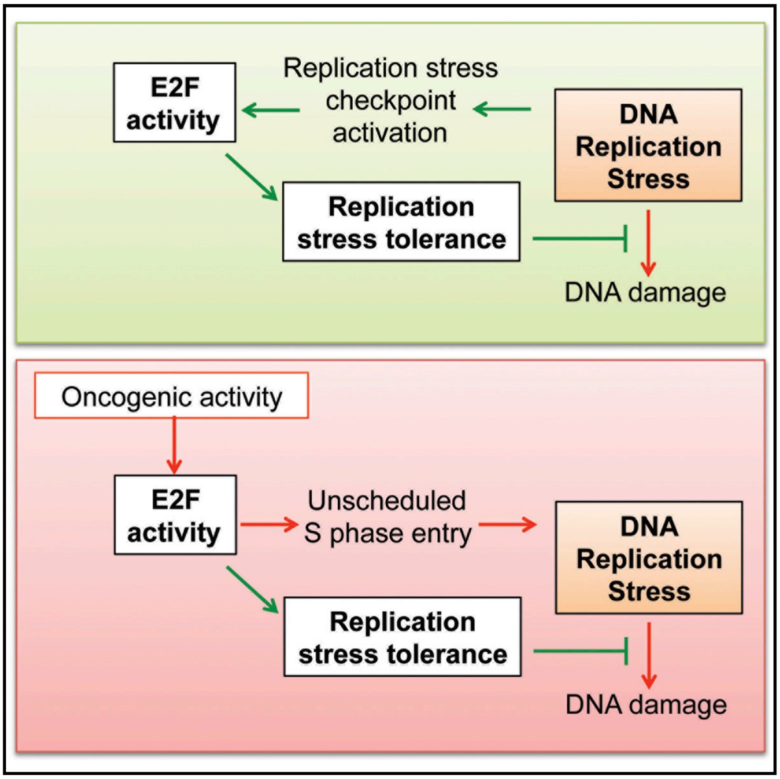

Highlights

- Gene expression plays an essential role in the response to replication stress
- Key stress response functions depend on sustained E2F-dependent transcription
- E2F activity is a key mechanism to cope with and recover from replication stress
- E2F activity limits DNA damage resulting from oncogene-induced replication stress

Authors

Cosetta Bertoli, Anna E. Herlihy, Betheney R. Pennycook, Janos Kriston-Vizi, Robertus A.M. de Bruin

Correspondence

r.debruin@ucl.ac.uk

In Brief

Bertoli et al. establish a far greater role for transcriptional control in determining the outcome of replication-stress-induced events than previously suspected. Their data predict a model in which cells that experience oncogene-induced replication stress become addicted to E2F-dependent transcription to cope with high levels of replication stress.

Bertoli et al., 2016, Cell Reports *15*, 1412–1422
May 17, 2016 © 2016 The Author(s)
http://dx.doi.org/10.1016/j.celrep.2016.04.036

Cell Reports
Report

Sustained E2F-Dependent Transcription Is a Key Mechanism to Prevent Replication-Stress-Induced DNA Damage

Cosetta Bertoli,[1,4] Anna E. Herlihy,[1,4] Betheney R. Pennycook,[1] Janos Kriston-Vizi,[1,3] and Robertus A.M. de Bruin[1,2,*]
[1]MRC Laboratory for Molecular Cell Biology
[2]The UCL Cancer Institute
[3]Bioinformatics Image Core (BIONIC)
University College London, London WC1E 6BT, UK
[4]Co-first author
*Correspondence: r.debruin@ucl.ac.uk
http://dx.doi.org/10.1016/j.celrep.2016.04.036

SUMMARY

Recent work established DNA replication stress as a crucial driver of genomic instability and a key event at the onset of cancer. Post-translational modifications play an important role in the cellular response to replication stress by regulating the activity of key components to prevent replication-stress-induced DNA damage. Here, we establish a far greater role for transcriptional control in determining the outcome of replication-stress-induced events than previously suspected. Sustained E2F-dependent transcription is both required and sufficient for many crucial checkpoint functions, including fork stalling, stabilization, and resolution. Importantly, we also find that, in the context of oncogene-induced replication stress, where increased E2F activity is thought to cause replication stress, E2F activity is required to limit levels of DNA damage. These data suggest a model in which cells experiencing oncogene-induced replication stress through deregulation of E2F-dependent transcription become addicted to E2F activity to cope with high levels of replication stress.

DNA replication stress (RS) is defined as inefficient DNA replication that causes DNA replication forks to progress slowly or stall, making them susceptible to DNA damage (Abraham, 2001; Jackson and Bartek, 2009; McGowan and Russell, 2004). RS can be caused by many factors like deregulation of components required for DNA synthesis, a decrease or increase in the frequency of replication initiation, and factors that block replication forks. The ability of cells to cope with RS is largely dependent on the action of the RS checkpoint, a conserved signaling pathway that constantly monitors for the loss of integrity of the DNA replication fork (Branzei and Foiani, 2010). RS leads to the accumulation of single-stranded DNA (ssDNA), which is coated by the ssDNA-binding protein complex replication protein A (RPA) and activates the sensor kinase ATR and its downstream effector kinase Chk1 (Cimprich and Cortez, 2008). The activation of this checkpoint aims to prevent DNA damage, a potential source of genomic instability. The RS checkpoint arrests cell-cycle progression, arrests and stabilizes on-going forks to prevent their collapse, blocks initiation of replication from late origins, and finally, when the stress is resolved, allows replication to resume. A large body of evidence supports a critical role for post-translational modifications, such as phosphorylation, sumoylation, and ubiquitination, in the RS checkpoint response (Huen and Chen, 2008; Jackson and Bartek, 2009). Whereas these regulatory events have been shown to be major determinants of checkpoint functions, little is known about the role of transcription in the cellular response to RS. Previous work from our lab has shown that E2F-dependent cell-cycle transcription is part of the checkpoint transcriptional response (Bertoli et al., 2013a), but the importance of this for specific checkpoint functions remains largely untested.

Transcriptional control during the G1 and S phases of the cell cycle depends on the E2F family of transcription factors in mammalian cells (Bertoli et al., 2013b). Activation of E2F-dependent transcription (from now on referred to as E2F transcription) is tightly regulated, as it controls the entry of cells into S phase and into the cell cycle. Under physiological conditions, it is driven by cyclin-dependent kinases that are activated downstream of growth factor signaling (Bertoli et al., 2013b). Oncogenes, such as Ras, c-Myc, and cyclin E, deregulate E2F-dependent G1/S transcription to drive passage into S phase and cell proliferation. By accelerating S phase entry, these oncogenes can generate RS (Hills and Diffley, 2014). Upon S phase entry, E2F transcription is inactivated via a negative feedback loop involving the transcriptional repressor E2F6, an E2F target itself (Bertoli et al., 2013a; Giangrande et al., 2004). Our previous work showed that, in response to RS, the checkpoint actively maintains E2F transcription via Chk1-dependent phosphorylation and inactivation of E2F6 (Bertoli et al., 2013a). Here, we provide evidence that sustained E2F transcription functions to maintain the expression of many proteins with key roles in the RS checkpoint response. The expression of E2F-dependent targets is not just required but sufficient for accomplishing crucial checkpoint functions such as stabilizing on-going replication forks and allowing replication to resume after the arrest. Importantly, we find that, in the

 © 2016 The Author(s)
This is an open access article under the CC BY-NC-ND license (http://creativecommons.org/licenses/by-nc-nd/4.0/).

OPEN ACCESS
CellPress

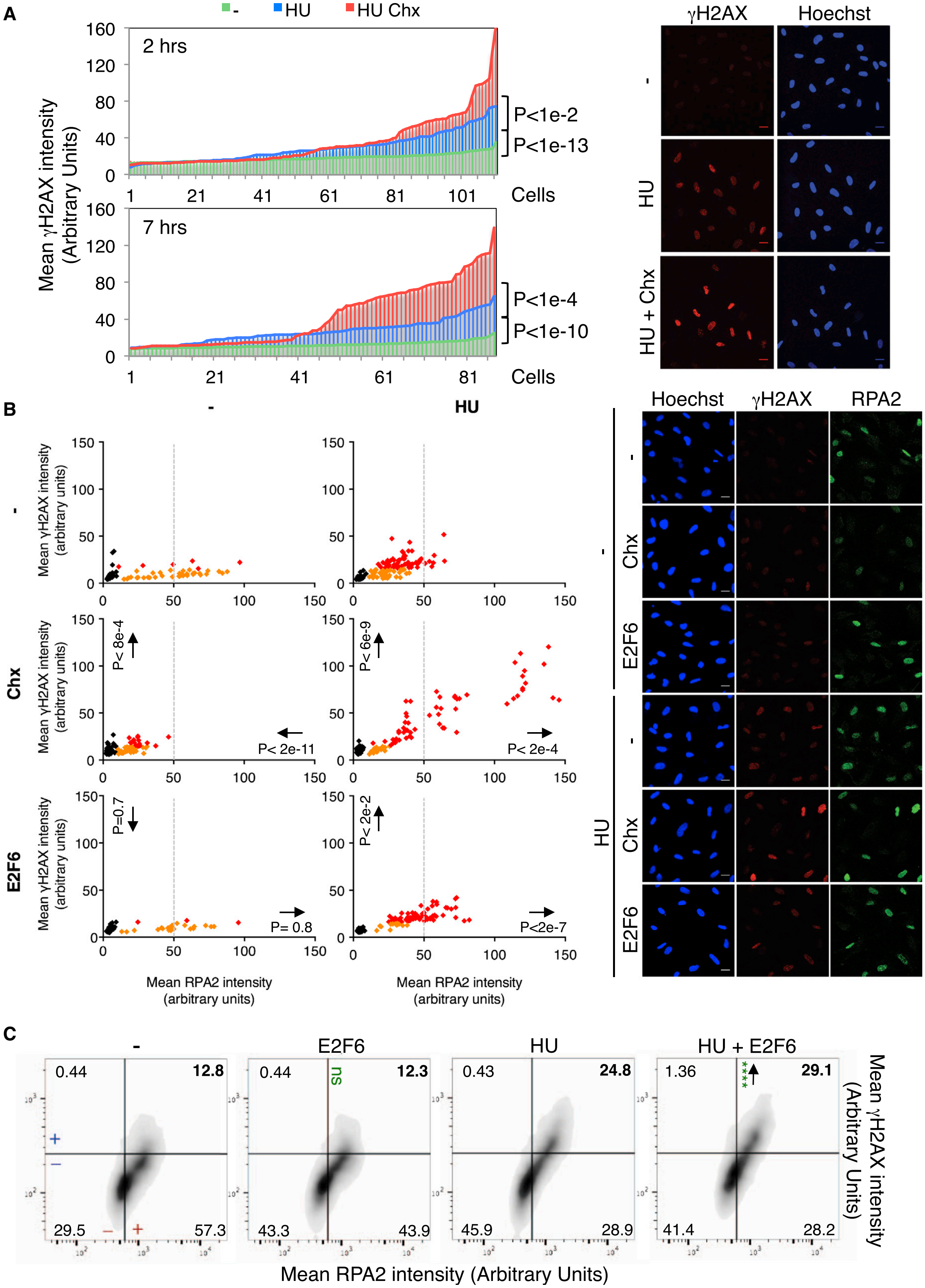

(legend on next page)

context of oncogene-induced RS, where increased E2F activity drives proliferation, which is thought to cause RS, paradoxically E2F transcription is required to limit DNA damage levels. Thus, E2F transcription is a key mechanism in the tolerance to RS.

RESULTS

E2F Transcription and Active Protein Synthesis Are Required to Prevent RS-Induced DNA Damage

Our previous work shows that, in human cells, maintaining E2F transcription is important to prevent apoptosis in response to RS (Bertoli et al., 2013a). However, how it contributes to RS tolerance remains unknown. In yeast, protein synthesis is not required for cell viability during the cellular response to RS (Pellicioli et al., 1999; Tercero et al., 2003). To test whether continuous expression of E2F target genes is important for RS response in human cells, we first tested whether de novo protein synthesis is necessary to prevent DNA damage following the cellular response to RS. RS was induced via acute treatment with hydroxyurea (HU), which depletes the pools of dinucleotide triphosphates (dNTPs) by inhibiting ribonucleotide reductase (RNR) activity and results in replication fork stalling. To assay the levels of DNA damage, we measured the intensity of H2AX hyperphosphorylation (γH2AX), which is a hallmark of ATM activity and therefore a read out for DNA damage. Intensity was measured by quantitative immunofluorescence of chromatin-bound γH2AX in single nuclei, similarly to Toledo et al. (2013). Both 2 and 7 hr HU treatment results in a significant increase in γH2AX signal when compared to untreated control cells, indicating the presence of some DNA damage in cells experiencing RS (Figure 1A). When the protein synthesis inhibitor cycloheximide (Chx), which blocks translation and prevents de novo protein synthesis, was added in addition to HU treatment the extent of DNA damage (γH2AX intensity) was significantly increased compared to HU treatment alone (Figure 1A). Thus inhibiting protein synthesis increases the extent of DNA damage induced by RS, suggesting a requirement for de novo protein translation during the response to RS in mammalian cells. To quantify levels of DNA damage resulting more specifically from RS, we then assessed the chromatin recruitment of ssDNA-binding protein replication protein A2 (RPA) alongside γH2AX. RPA coats the extended amounts of ssDNA that occur during RS (Zou et al., 2006) and is used as an indicator of RS. We analyzed by quantitative immunofluorescence the intensity of both chromatin-bound RPA (marker of RS) and γH2AX (marker of DNA damage) in individual S phase nuclei; this allows us to analyze the extent of RS-induced DNA damage. These data show that the increase in DNA damage (γH2AX) seen following 7 hr Chx and HU treatment is highest in cells labeled with RPA, indicating that the DNA damage is resulting from RS (Figures 1B and S1A). Importantly, Chx alone does not generally cause an increase in γH2AX signal (Figures S1B and S2A). These findings indicate that sustained protein synthesis is required to prevent the occurrence of RS-induced DNA damage.

Next, we tested the contribution of sustained E2F transcription in preventing RS-induced DNA damage. Doxycycline-induced overexpression of the repressor E2F6 interferes with the checkpoint-dependent maintenance of E2F transcription (Figure S1C); preventing this response allows the study of its role following RS. If sustained E2F transcription is involved in the tolerance to RS then overexpression of the repressor E2F6, and subsequent loss of E2F transcription, would be expected to result in increased levels of DNA damage following HU-induced RS. As before, the intensity of chromatin-bound γH2AX and RPA in individual S phase nuclei was measured after 7 hr HU treatment. E2F6 overexpression was induced with a short 2 hr pre-treatment of Doxycycline in HU-treated or untreated cells. Results reported in Figures 1B, S2A, and S2B show an increase in γH2AX labeling upon E2F6 overexpression in HU treatment compared to HU treatment alone in both HEK293 TRex E2F6 and RPE1 TetON E2F6 cells. This increase is seen in nuclei with high levels of RPA, indicating this is RS-induced DNA damage. E2F6 overexpression in untreated cells does not cause an increase in γH2AX levels. As expected, because only the E2F-dependent RS transcriptional response is compromised, the increase in γH2AX signal is less pronounced than that seen in Chx-treated cells. To confirm these results, we increased the throughput of the assay by adopting a protocol for fluorescence-activated cell sorting (FACS) analysis, based on Forment et al. (2012). This method provides a more quantitative way of measuring differences in both γH2AX and RPA staining in higher numbers of individual cells. This analysis confirms our results showing a significant increase in γH2AX with E2F6 overexpression in cells treated with HU but no significant change in untreated cells (Figure 1C). Increased DNA damage in E2F6-overexpressing cells in HU was also confirmed by western blot of whole-cell extract (WCE) (Figure S2C). These findings suggest that sustained E2F transcription is required to prevent RS-induced DNA damage in human cells.

Protein Synthesis and E2F Transcription Are Required to Maintain the Levels of Checkpoint Proteins

Our results suggest a role for active protein synthesis and specifically E2F transcription in the cellular response to RS. Because E2F cell-cycle target genes include most of the major DNA replication, repair, and checkpoint effectors, we hypothesized that

Figure 1. E2F Transcription and Active Protein Synthesis Are Required to Prevent RS-Induced DNA Damage

(A) Graph of mean chromatin-bound γH2AX intensity of single nuclei. Treatment and times are as shown for RPE1 cells. p, significant differences of S phase cells with Wilcoxon. Representative images are from 7 hr. The scale bar represents 20 μm.

(B) Scatterplot of mean intensity of chromatin-bound RPA2 versus γH2AX of single nuclei. Treatments shown for 7 hr for RPE1 TetON E2F6. E2F6 overexpression, Doxy. Black, non-S phase cells (RPA2 < 10 a.u.); orange and red dots, low and high levels of γH2AX, respectively (arbitrary threshold γH2AX = 15 a.u.). p, differences on both axes of S phase cells with Wilcoxon compared to control (−/HU as appropriate). Arrows show change in mean. Representative images are shown. The scale bar represents 20 μm.

(C) Density plot of FACS for RPA2 versus γH2AX intensity; treatments shown are for 7 hr for RPE TetON E2F6 cells. Doxy, 2 μg/ml. Quadrants define RPA2 or γH2AX −/+ cells (−/+ in red and blue, respectively); percentage of cells is shown. Around 10,000 cells were collected per condition. Logarithmic scale identical for all. ****$p < 0.0001$ compared to non-E2F6 control; Student's t test. Arrows show change of mean.

See also Figures S1 and S2.

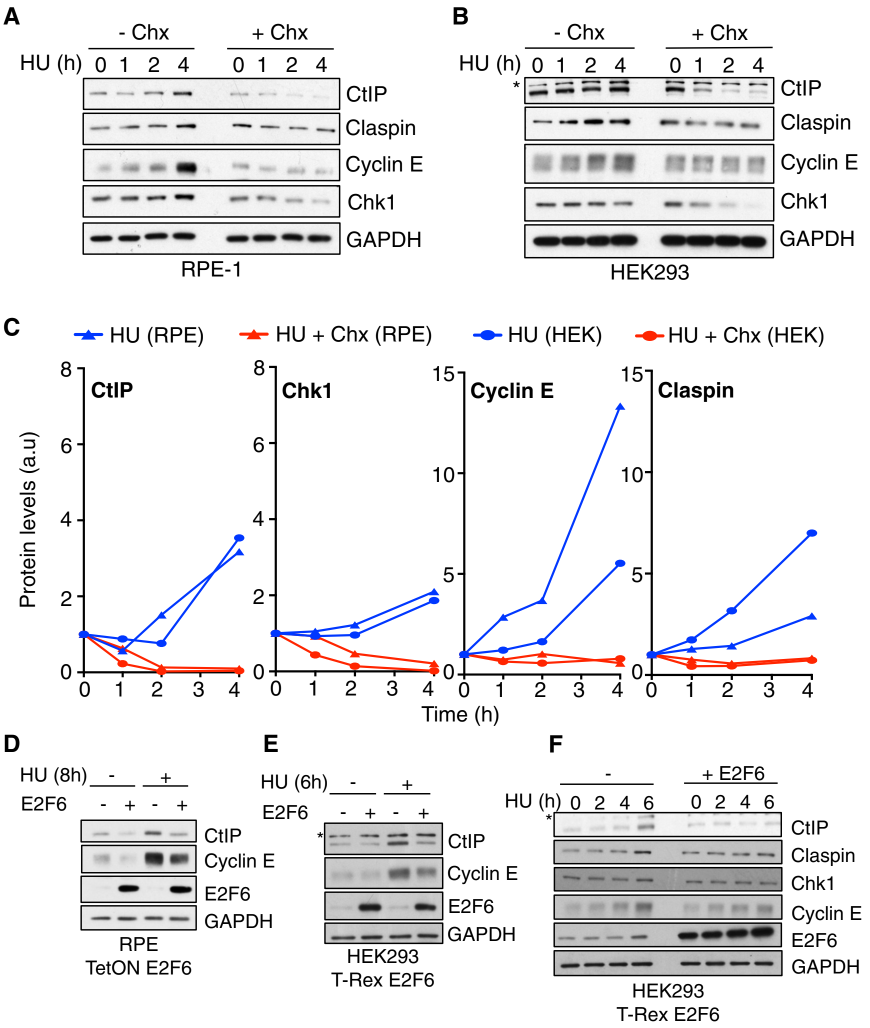

Figure 2. E2F Transcription and Active Protein Synthesis Are Required to Maintain and/or Upregulate the Levels of Checkpoint Proteins

Asterisks mark unspecific bands.

(A) Western blot of WCE (whole-cell extract), RPE1 cells, treatment, and times shown.

(B) Western blot of WCE, HEK293 cells, treatment, and times shown.

(C) Quantification of (A) (RPE1) and (B) (HEK293), normalized to GAPDH and 0 hr.

(D) Western blot of WCE, RPE1 TetON E2F6 cells, treatment, and times shown. E2F6 overexpression, Doxy.

(E) Western blot of WCE, HEK293 T-Rex E2F6 cells, treatment, and times shown.

(F) Western blot of WCE, HEK293 T-Rex E2F6 cells, treatment, and times shown.

See also Figure S3.

active protein synthesis prevents RS-induced DNA damage by maintaining the levels of these proteins. To assess this, we analyzed the stability of a number of key checkpoint proteins during RS in HEK293, RPE1, and T98G cells (Figures 2A–2C, S3A, and S3B). As expected, these checkpoint proteins, which are all E2F targets, are upregulated during HU-induced RS. The addition of the translational inhibitor Chx reveals two types of proteins. (1) The first are proteins for which ongoing protein synthesis is required to significantly increase their abundance in response to RS. These proteins are relatively stable, and Chx addition only prevents the HU-induced accumulation but does not result in a loss of protein abundance, cyclin E, and claspin. (2) The second are proteins that are inherently unstable and so Chx treatment results in a dramatic loss of their abundance, CtIP, and Chk1. For these proteins, active protein synthesis during RS is mainly required to maintain their levels rather than to significantly increase abundance. Interestingly, this group includes checkpoint proteins that show increased degradation rates during the checkpoint response (Figure S3A), suggesting they are targeted for degradation in a checkpoint-dependent manner, as previously reported for Chk1 (Zhang et al., 2005).

Overall, these data support the hypothesis that, to correctly regulate the level and activity of crucial checkpoint effector proteins, cells require active protein synthesis during RS.

Protein abundance is, among others, a function of both transcript levels (a function of transcription and mRNA stability) and protein stability. Our data suggest an important role for transcription, specifically E2F transcription, in preventing RS-induced DNA damage. To establish whether active transcription is required for maintaining protein levels during RS, we treated cells with the transcriptional inhibitor actinomycin D. In response to HU, protein levels were affected similarly by transcriptional and protein synthesis inhibition (Figure S3C), indicating that active transcription is required for maintaining optimal levels of proteins during RS. The same effect on protein levels was seen when just E2F transcription was inhibited, through doxycycline-induced overexpression of the repressor E2F6. As seen when inhibiting transcription or translation, preventing E2F transcription by E2F6 overexpression during RS results in a lower abundance of key checkpoint effector proteins in both HEK293 TRex E2F6 and RPE1 TetON E2F6 cells (Figures 2D–2F). These data suggest that sustained E2F transcription is required for maintaining optimal levels of key checkpoint proteins during the cellular response to RS.

Sustained E2F Transcription Is Necessary for the Arrest and Stabilization of Replication Forks

Our results indicate that sustained E2F transcription is required for the cellular response to RS. We therefore investigated which specific biological processes essential to the RS checkpoint response require sustained E2F transcription. An important process to prevent RS-induced DNA damage involves the arrest and stabilization of ongoing replication forks (Seiler et al.,

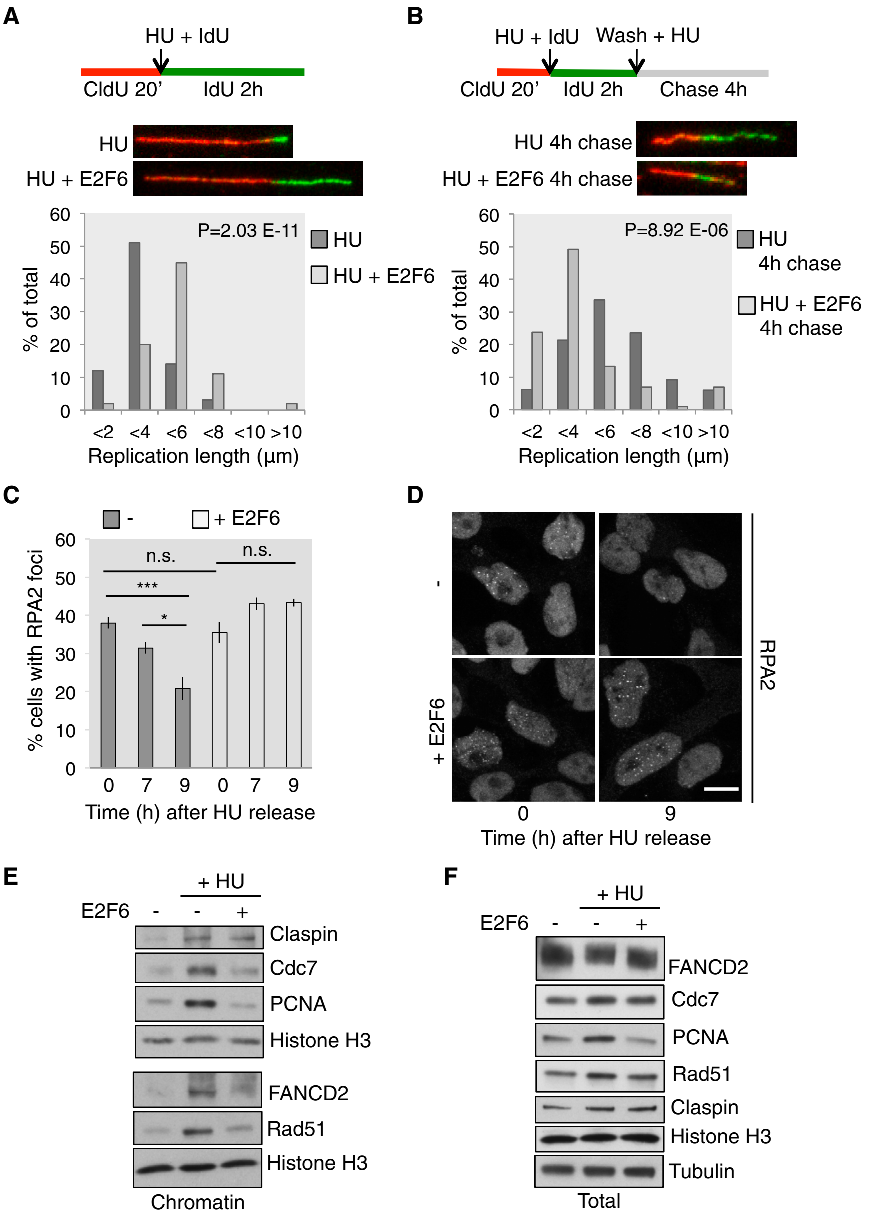

Figure 3. Sustained E2F Transcription Is Necessary for Checkpoint Functions

(A) DNA fiber analysis schematic and representative images of individual fibers, HEK293 T-Rex E2F6 cells. Bar graphs of green track length, 2 hr HU −/+ E2F6 (Doxy 2 µg/ml). p, significantly longer tracks with E2F6; Student's t test.

(B) Schematic and representative images, HEK293 T-Rex E2F6 cells. Bar graphs of green track length, 4 hr chase in HU −/+ E2F6 (Doxy 2 µg/ml). p, significantly shorter tracks with E2F6; Student's t test.

(C) Bar graph of % cells with RPA2 foci at times shown after release from 16 hr HU −/+ E2F6 (Doxy 2 µg/ml). n = 3; >130 for each; error bars = SEM. ***p < 0.001; *p < 0.05 with ANOVA and Tukey's.

(D) Representative images for (C). The scale bar represents 10 µm and is the same for all.

(E) Western blot of chromatin preparation, RPE1 TetON E2F6 cells, 7 hr, treatments shown. E2F6, Doxy 2 µg/ml.

(F) Western blot of WCE, RPE1 TetON E2F6 cells, 7 hr, treatments shown. E2F6, Doxy 2 µg/ml.

See also Figure S3 and Movies S1 and S2.

2007). DNA fiber analysis was used to evaluate the arrest of ongoing forks during HU treatment (Lossaint et al., 2013), with and without active E2F transcription. DNA replication tracks were labeled with the nucleotide analog CldU (red) and then HU was added with the second analog IdU (green; Figure 3A). These nucleotide analogs are incorporated during ongoing replication and can then be visualized. The length of the green tracks displayed in the bar graph represents replication progression during RS. As expected, HU treatment alone induces a replication arrest, resulting in a population of short tracks. Strikingly, cells unable to sustain E2F transcription (+E2F6) as part of their checkpoint response continue to replicate their DNA further during HU treatment than control cells (Figure 3A). Importantly, overexpression of E2F6 in untreated cells does not affect the length of DNA tracks at these time points (Figure S3D). These data suggest that active E2F transcription is required to efficiently arrest replication forks in response to RS.

We then assessed the role of E2F transcription in stabilizing stalled replication forks during RS. Previous work has shown that an inability to stabilize replication forks during RS, over time, results in shorter DNA tracks in fiber analysis likely due to resection of the newly synthesized DNA (Lossaint et al., 2013; Schlacher et al., 2011). DNA fiber analysis was carried out in HU-treated cells with wild-type or overexpressed levels of E2F6. After the IdU pulse (green), the cells were chased in HU in the absence of nucleotide analogs for 4 hr (Figure 3B). If stalled replication forks are stable, the green tracks do not shorten; however, if the forks are not properly stabilized, this can result in shorter tracks, likely due to unchecked nuclease activity. The length of the green (IdU) tracks during the chase period is significantly reduced in E2F6 overexpressing cells (HU + E2F6) compared to control (HU; Figure 3B), suggesting that the maintenance of fork stability is compromised in the absence of a proper transcriptional response. Based on these results, we conclude that sustained E2F transcription is important for replication fork arrest and stabilization in response to RS.

Protection and Resolution of Stalled Replication Forks Requires E2F Transcription

Another important function of the RS checkpoint response is to resolve arrested replication forks to allow replication to resume

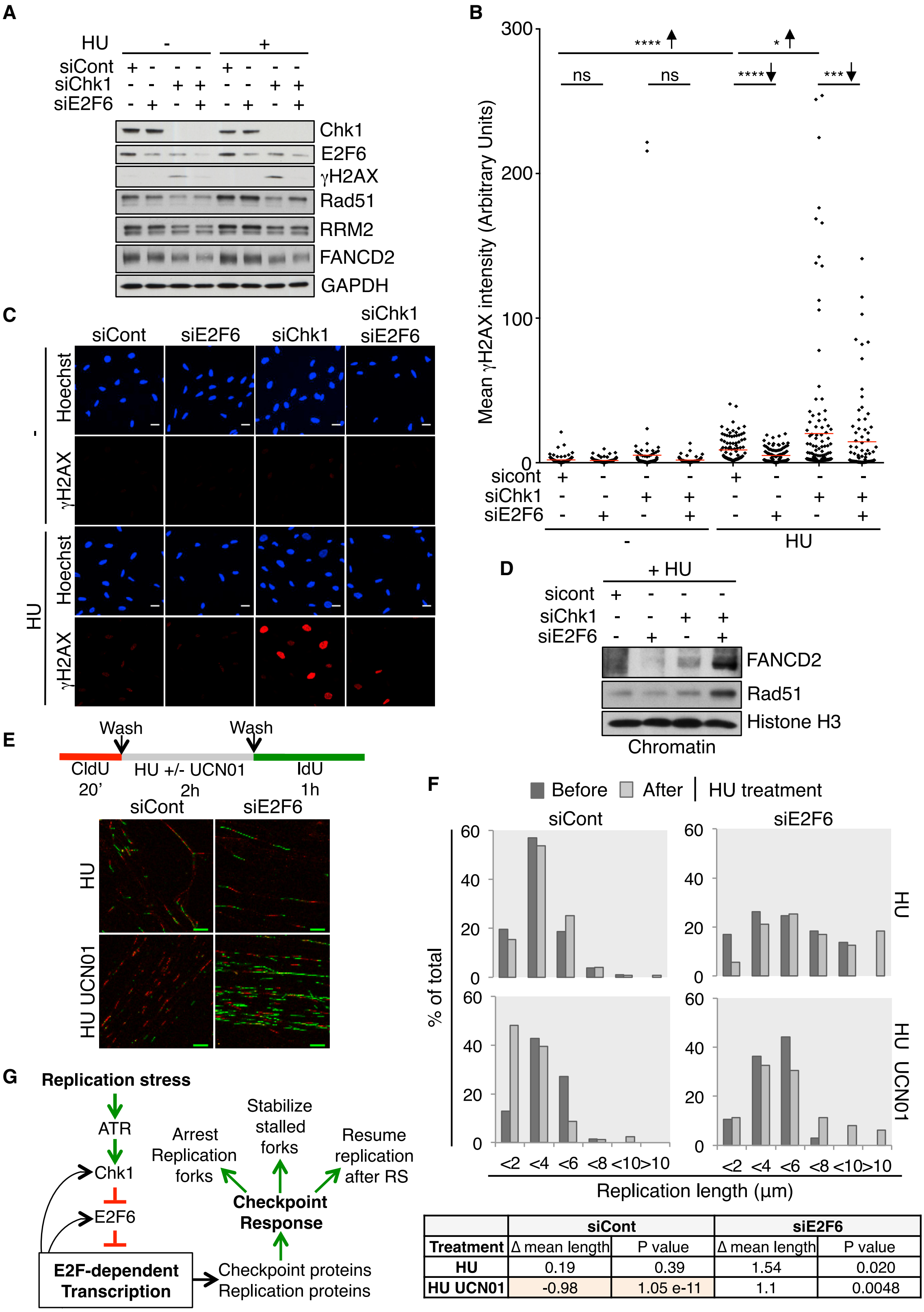

	siCont		siE2F6	
Treatment	Δ mean length	P value	Δ mean length	P value
HU	0.19	0.39	1.54	0.020
HU UCN01	-0.98	1.05 e-11	1.1	0.0048

(legend on next page)

once the stress has been relieved (Petermann and Helleday, 2010). This can be assessed by monitoring the number of cells containing RPA2 foci, which indicate stalled replication forks, at various times after release from HU-induced RS. To test whether there might be a role for E2F transcription in this process, cells were treated with HU with or without E2F6 overexpression and then washed and released into normal medium. After HU treatment (0 hr after release), control and +E2F6 show a similar percentage of cells containing RPA2 foci, indicating a similar number of cells in S phase and experiencing RS, excluding any cell-cycle effects of E2F6 overexpression (Figures 3C and 3D). After release from HU, control cells resolved RPA2 foci, as seen by a lower percentage of cells containing foci 7 and 9 hr after release. However, cells released from HU+E2F6, which were unable to sustain E2F transcription during RS, were unable to resolve arrested forks as indicated by the maintenance of high levels of RPA2 foci. To test this idea further, we monitored RPA2 foci resolution after HU release by in vivo time-lapse imaging, exploiting a HEK293 cell line stably expressing GFP-RPA2 (Movies S1 and S2). The inhibition of E2F transcription during HU treatment strongly delayed the resolution of RPA2 foci, confirming the previous results.

A critical step in the checkpoint response is the formation of a protective complex at stalled replication forks that enables fork stalling, stabilization, and restart (Branzei and Foiani, 2010). We therefore investigated whether sustained E2F transcription is required for the recruitment of factors involved in this process to chromatin. Strikingly, protective factors such as Rad51, FANCD2 (Lossaint et al., 2013), PCNA (Ciccia and Elledge, 2010), and Cdc7 (Yamada et al., 2013, 2014), which become bound to chromatin upon 7 hr HU treatment, show defective recruitment to chromatin when transcription is blocked by E2F6 overexpression (Figure 3E). Whereas overexpression of E2F6 during HU treatment reduces the levels of some of these proteins compared to HU treatment alone, they can still be detected in total lysate (Figure 3F). This shows that E2F transcription is required for the formation of a protective complex at forks during RS. Overall, these data show that sustained E2F transcription is required for specific checkpoint functions—replication fork stalling, stability, and protection during RS and resolving stalled forks after the arrest.

E2F Activity Is a Key Mechanism of the Checkpoint Response to Prevent DNA Damage

To determine whether maintaining expression of E2F targets is a key mechanism of the checkpoint response, we tested whether sustaining E2F transcription alone (by silencing the repressor E2F6) could rescue checkpoint functions in cells with a compromised checkpoint response (by silencing the checkpoint effector kinase Chk1). We first assessed whether activating E2F transcription (siE2F6) could prevent DNA damage from accumulating in checkpoint-compromised cells (siChk1), in response to 4 hr HU treatment. In cells treated with siChk1 and HU-induced RS, restoring E2F transcription (siE2F6) can indeed significantly reduce DNA damage levels (γH2AX) as detected by western blot (Figures 4A and S4A) and quantitative immunofluorescence analysis in individual cells (Figures 4B and 4C). Importantly, the significant decrease in γH2AX signal is detected in RPA-labeled cells, and with a second siRNA targeting E2F6 (siE2F6-2) (Figures S4B and S4C). These data indicate that E2F transcription can reduce RS-induced DNA damage levels in cells with a compromised checkpoint response.

We next assessed whether activating E2F transcription (siE2F6) could also prevent DNA damage in response to HU treatment in cell depleted for ATR. ATR phosphorylates Chk1 in response to RS, but Chk1 can be activated when ATR is compromised (Buisson et al., 2015), and there are also ATR-dependent and Chk1-independent aspects of the RS response (Koundrioukoff et al., 2013). Following 4 hr HU, siATR-treated cells accumulate less γH2AX signal in RPA-labeled cells compared to that observed in siChk1-treated cells. However, we still observe a small but significant reduction in both γH2AX and RPA signal when E2F6 is co-depleted during HU treatment (Figures S4D and S4F), confirming the results obtained with Chk1. Furthermore, the co-depletion of E2F6 and ATR reduces the extent of RPA2 phosphorylation, another marker of ATM activity (Figure S4E). Overall, these results indicate that restoring E2F transcription can reduce RS-induced DNA damage in checkpoint-compromised cells.

Given the reported role of RNR enzyme in preventing RS, we evaluated the protein levels of the RNR subunit RRM2, a well-known E2F target. An increase in RRM2 has been recently shown to counteract RS and DNA damage induced by the inactivation of the checkpoint proteins ATR and Chk1 (Buisson et al., 2015). The levels of RRM2 increase in response to 4 hr HU (Figure S4E), according to our previous findings (Bertoli et al., 2013a), and the depletion of Chk1 decreases the levels of RRM2 (Figure 4A), supporting a role of Chk1 in maintaining RRM2 levels. Most importantly, the levels of RRM2 do not change following E2F6 depletion (Figures 4A and S4E), suggesting that the protective effect of

Figure 4. E2F Activity Is Sufficient to Prevent DNA Damage and Rescue Checkpoint Functions in Cells with a Compromised Checkpoint Response

(A) Western blot of WCE, RPE1 cells transfected with siRNA shown −/+ 4 hr HU,.

(B) Column scatter graph of mean intensity of γH2AX of individual nuclei, RPE1 cells from 4A. Transfected with siRNA are shown −/+ 4 hr HU. Mean is in red. ****$p < 0.0001$; ***$p < 0.001$; *$p < 0.05$ with Wilcoxon. Arrows show change of mean.

(C) Representative images for (B). The scale bar represents 20 μm and is the same for all.

(D) Western blot of chromatin preparation, RPE1 cells transfected with siRNA shown, HU 4 hr.

(E) Schematic and representative images for (F), T98G cells. The scale bar represents 10 μm.

(F) Bar graphs of length of DNA tracks before and after HU treatment with siRNA and treatments shown. Table is of differences in mean length (after-before). p, difference with Wilcoxon.

(G) RS checkpoint activation sustains E2F transcription. This transcriptional response is essential and sufficient during RS to maintain optimal protein levels and important functions of the checkpoint response to prevent RS-induced DNA damage.

See also Figures S4 and S5.

E2F6 depletion does not derive from increased levels of deoxyribonucleotides.

Next, we assessed whether activating E2F transcription (siE2F6) allows the replication fork protective complex to form in response to RS in checkpoint-compromised cells (siChk1). Control silenced cells do not yet show strong signs of RS after 4 hr HU treatment (Figures 4A, 4B, and S4C) and therefore display limited Rad51 and FANCD2 recruitment to chromatin (Figure 4D). In contrast, cells depleted of Chk1 (siChk1) experience high levels of RS-induced DNA damage in response to 4 hr HU treatment but limited protective complex formation. Strikingly, the inactivation of E2F6 in checkpoint-compromised cells (siE2F6 siChk1) can drive chromatin recruitment of FANCD2 and Rad51 (Figure 4D), correlating with the reduction in RS-induced DNA damage levels seen (Figures 4B and S4C). These data indicate that maintaining E2F transcription is required and sufficient to prevent the accumulation of RS-induced DNA damage, likely though driving the formation or maintenance of a protective complex at replication forks.

Next, we investigated whether maintaining E2F transcription, in cells with a compromised checkpoint response, is sufficient for resuming replication once the stress has been removed. DNA fiber analysis incorporating CldU was used to measure the length of DNA tracks, indicating the progression of DNA replication. Cells were then treated with 2 hr HU to cause RS, which is known to arrest replication. Then cells were washed and released into normal medium containing the IdU nucleotide analog and the length of DNA tracks measured. This shows the cells ability to resume replication following release from a period of RS. Control cells show similar track lengths before and after HU treatment (Figures 4E and 4F), showing they are able to resume replication following HU-induced RS. However, cells treated with a Chk1 inhibitor (UCN01), and therefore checkpoint-compromised, have significantly shorter tracks after HU treatment, indicating they are impaired in resuming replication. Strikingly, in this same checkpoint-compromised set-up (HU+UCN01) maintaining E2F transcription, via E2F6 depletion (siE2F6 or siE2F6-2) restores the cells' ability to restart DNA replication following RS (Figures 4E, 4F, and S5A). These data suggest that sustained E2F transcription is sufficient, even without a proper checkpoint response, for the formation of a protective complex at stalled replication forks to allow replication to resume after HU treatment and to prevent DNA damage. Overall, these data suggest that sustained E2F transcription is an essential part of the checkpoint response mechanism to RS (Figure 4G).

Tolerance to Oncogene-Induced RS Requires E2F Transcription

Oncogenes, such as Ras, c-Myc, and cyclin E, induce E2F transcription to drive entry into S phase and cell proliferation. The generally accepted view is that this unscheduled S phase entry is at the likely basis of oncogene-induced RS, suggesting a direct link between E2F induction and oncogene-induced RS (Hills and Diffley, 2014). However, our data show that sustained E2F transcription is essential for key checkpoint mechanisms to prevent RS-induced DNA damage. This suggests that the upregulation of E2F transcription might inadvertently contribute to tolerance to oncogene-induced RS by preventing DNA damage. To investigate this possibility, we tested whether maintaining E2F transcription is required to prevent DNA damage caused by oncogene-induced RS. We created a stable, E2F6-inducible RPE1 cell line in which we can also induce oncogenic Myc activity (RPE1 TetON E2F6 c-Myc-ER). Importantly, the levels of RS induced by c-Myc-ER induction are comparable to the levels induced by HU treatment in our experimental conditions (Figures 5A and S5C), indicating these are within a physiologically relevant range. This cell line allows the separate induction of c-Myc with hydroxytamoxifen (4OH-T) to cause oncogene-induced RS (Figure S5B) and inducible overexpression of E2F6 with doxycycline to repress E2F-transcription. Taking CtIP as a representative E2F target we see accumulation following c-Myc induction, which is prevented by E2F6 overexpression (Figure 5B). As before, we analyzed γH2AX intensity in single S phase nuclei as a measure of DNA damage. Overexpression of E2F6 alone does not result in a significant increase in γH2AX labeling in S phase cells (Figure 5D). However, overexpression of E2F6 in the presence of c-Myc induction (c-Myc/E2F6) significantly increases DNA damage during S phase compared to c-Myc induction alone (c-Myc), as indicated by increased γH2AX labeling (Figure 5D). In accordance with this, overexpression of E2F6 decreases the survival of cells following c-Myc induction, indicating that E2F transcription is required for cell survival following oncogene-induced RS (Figure S5D). These data suggest that tolerance to oncogene-induced RS depends on E2F activity.

Next, we tested whether, in response to oncogene-induced RS, as in response to HU, chromatin recruitment of fork-stabilizing proteins was impaired in the presence of E2F6 overexpression. c-Myc-induced RS results in the recruitment of CtIP and FANCD2 to chromatin. This is impaired by concurrent overexpression of E2F6 (Figure 5C), indicating that E2F transcription is also required in the response to oncogene-induced RS for the recruitment of fork-stabilizing proteins. Finally, we tested if a decrease in the repressor protein E2F6, and hence an increase in E2F transcription would reduce the levels of RS-induced DNA damage caused by oncogenic activity (c-Myc). c-Myc induction causes an increase in the levels of γH2AX. An increase in E2F transcription (siE2F6) reduced the levels of RS-induced DNA damage caused by oncogenic activity (Figures 5E and S5E). Together, these data support an important role for E2F transcription in the tolerance to oncogene-induced RS (Figure 5F).

DISCUSSION

The work presented here establishes that sustained E2F transcription is a key mechanism in the RS checkpoint response (Figure 4G). Our work shows that sustained E2F transcription is both required and sufficient for many essential functions of the checkpoint response, including fork stalling, stabilization, and resolution. Our data suggest that critical checkpoint proteins require sustained E2F transcription, which is actively maintained in a checkpoint-dependent manner, to correctly regulate their levels and likely activity during RS. Indeed, many of the proteins with critical roles in the stalling and stabilization of forks and restart of replication, including Brca1, Brca2, Rad51, CtIP, and BLM

OPEN ACCESS
CellPress

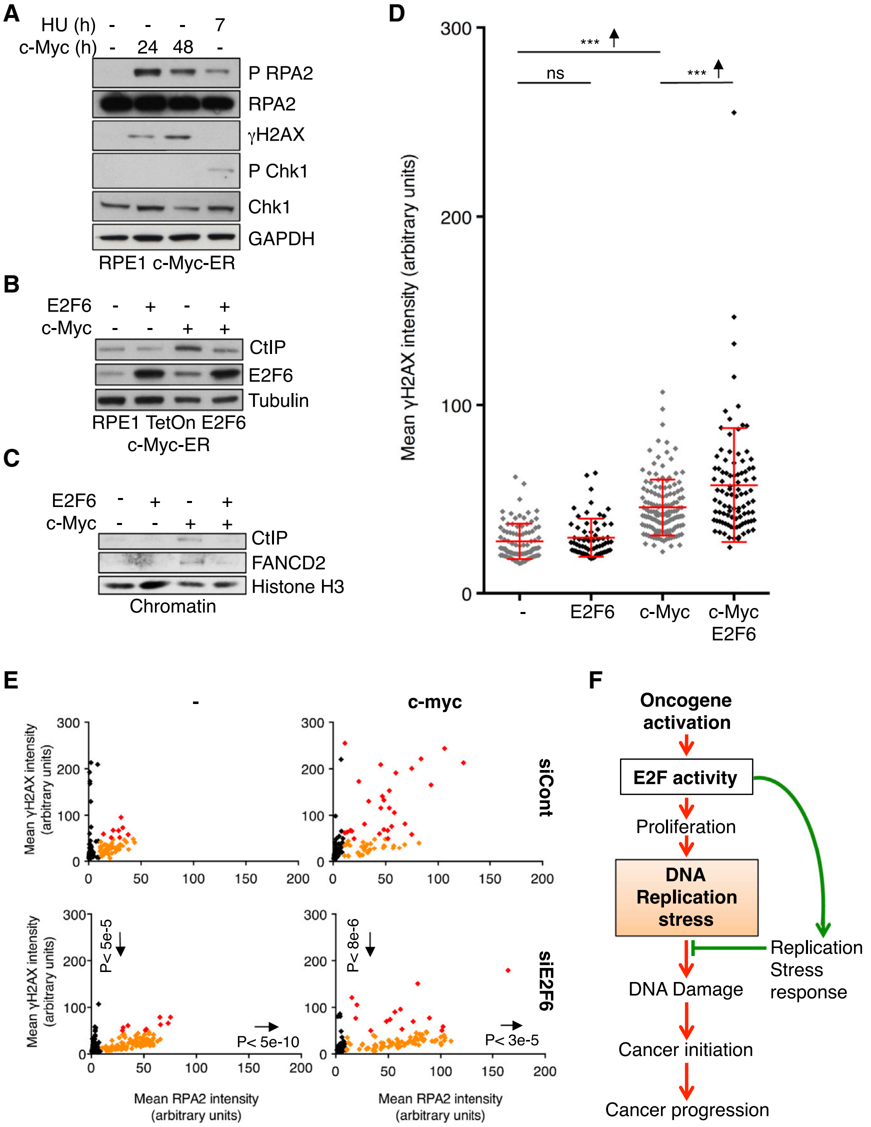

Figure 5. E2F Activity Is Required for Tolerance to Oncogene-Induced RS

(A) Western blot of WCE, RPE1 c-Myc-ER cells. Treatment and times shown.

(B) Western blot of WCE, RPE1 TetON E2F6 c-Myc-ER cells treatments shown, 72 hr. c-Myc, 4OH-T 200 nM; E2F6, Doxy 1 μg/ml. CtIP is representative E2F target.

(C) Western blot of chromatin preparation of RPE1 TetON E2F6 c-Myc-ER cells −/+ c-Myc-ER 4OH-T, 100 nM, 48 hr, E2F6 overexpressed in the last 24 hr (Doxy, 2 μg/ml) as shown. Histone H3 is loading control.

(D) Column scatter graph (mean and SD in red) of mean γH2AX intensity in single S phase nuclei, RPE1 TetON E2F6 c-Myc-ER cells −/+ c-Myc-ER 4OH-T, 100 nM, 72 hr, E2F6 overexpressed in the last 24 hr (Doxy, 2 μg/ml) as indicated. Only S phase cells are shown (RPA2 > 20 a.u.). p, significant difference with Wilcoxon. ***$p < 0.001$. Arrows show change of mean.

(E) Scatterplot of mean intensity of RPA2 versus γH2AX of single nuclei, RPE1 TetON E2F6 c-Myc-ER cells transfected with siRNA shown −/+ c-Myc-ER induction, 48 hr. Black dots, non-S phase cells (RPA2 < 10 a.u.); orange and red, low and high levels of γH2AX, respectively (arbitrary threshold γH2AX = 50 a.u.). p, differences in S phase cells on both axes with Wilcoxon compared to appropriate siCont control. Arrows show change of mean.

(F) Sustained E2F transcription is a key mechanism in the RS checkpoint response. In the context of oncogene-induced RS, whereas increased E2F activity lies at the basis of causing RS, it is also responsible for limiting DNA damage levels resulting from RS.

See also Figure S5.

(Petermann and Helleday, 2010), are E2F targets. Sustained E2F transcription during RS is required for the increase of certain checkpoint protein, whereas, for others, it is needed to maintain their levels due to their short half-lives. Importantly, we also find that, in the context of oncogene-induced RS, where increased E2F activity is thought to cause RS, E2F transcription is required to limit DNA damage levels (Figure 5F).

Maintaining the expression of E2F targets in response to RS requires the checkpoint kinase Chk1 inhibiting a negative feedback loop involving the transcriptional repressor E2F6 to allow E2F transcription. As E2F6 and Chk1 are both E2F targets, E2F6 is able to repress both, allowing for the rapid downregulation of transcriptional and checkpoint responses once the checkpoint has been satisfied. This, in combination with the short half-life of some critical checkpoint proteins, would allow for robust inactivation of the checkpoint response once the stress has been dealt with to allow replication to resume. Currently, the mechanism that signals for the cell to resume replication is largely unknown. We propose that this particular network wiring, with coordinated control of transcription/translation and protein degradation, could enable the cell to quickly re-adjust the proteome once the stress has been resolved. Potentially, newly synthesized un-phosphorylated components replacing phosphorylated proteins at the replication fork could signal replication restart when the checkpoint is satisfied. We propose that the interplay between transcription, protein stability, and phosphorylation is important to regulate the level and activity of proteins involved in the highly dynamic chain of events that characterizes the checkpoint response and its resolution, opening a new perspective for future research.

In addition to a possible role in the mechanism signaling the cell to resume replication, robust inactivation of E2F transcription once the stress has been removed might also be important to prevent genomic instability. The defects caused by persistent E2F transcription during S phase remain to be established. However, genomic instability has been associated with transcription interfering with DNA replication (Helmrich et al., 2011), so

maintaining transcription of a large number of genes during DNA replication might result in genome instability. In addition, checkpoint and replication control proteins might cause genomic instability if their activity is not restrained when the cell resumes replication.

RS is a common feature of cells with activated oncogenes or absent tumor suppressors that accelerate the rate of S phase entry and disrupt the DNA replication schedule (Hills and Diffley, 2014). The ensuing RS-induced DNA damage triggers activation of the checkpoint kinases (ATM and Chk2). This induces senescence or apoptosis, serving as an initial barrier to inhibit tumor development in its early stages by preventing proliferation. It is hypothesized that persistent RS creates an environment that selects for mutations that bypass checkpoint functions (most notably p53) and rescues proliferation. This allows tumor progression, which generates DNA damage contributing to rapid evolution and heterogeneity in tumors. Oncogenes, such as Ras, c-Myc, and cyclin E, induce E2F transcription to drive passage into S phase and cell proliferation. The generally accepted view is that this unscheduled S phase entry is at the basis of oncogene-induced RS, suggesting a direct link between E2F induction and oncogene-induced RS. However, our data show that sustained E2F transcription is a key mechanism of the checkpoint response to prevent RS-induced DNA damage. Our work supports a model where an increase in E2F activity promotes oncogene-driven cellular proliferation causing RS but also provides the mechanism of RS tolerance, which allows the cells to cope with the increased rates of replication. The increased reliance on sustained E2F transcription creates a potentially large therapeutic window for damaging cancer cells experiencing RS without affecting normal cells.

EXPERIMENTAL PROCEDURES

Cell Culture and Transfection

Cell lines used were T98G, RPE-1 hTERT, and HEK293 T-Rex E2F6 (previously described; Bertoli et al., 2013a). RPE1 TetON E2F6 and RPE1 TetON E2F6 c-Myc-ER were created for this study. See the Supplemental Information for details of maintenance and creation of cell lines. Transfections were performed with Lipofectamine 2000 (Invitrogen) according to manufacturer instruction. See the Supplemental Information for siRNA sequences. Transfected cells were split 24 hr after transfection and then used 24 hr later for the experiments (−/+HU 4 hr) or for control western blot; these originate from the same transfection.

Treatments

HU, 0.5 mM. Doxycycline, 1 or 2 hr pretreat before HU, 4 μg/ml, unless otherwise stated. Chx, 10 min pretreat, 10 μg/ml. UCN01, 100 nM. Actinomycin D, 10 μg/ml. 4OH-T (Sigma-Aldrich), 100 nM, unless otherwise stated.

Immunofluorescence

Cells were pre-extracted for 1 min in ice cold PBS 0.2% Triton X-100 (0.15% for HEK293), fixed in 4% formaldehyde for 20 min, and processed similarly to Toledo et al. (2013). Images were obtained with Leica TCS SP5 or SPE2 63× objective lens, processed in Fiji. See the Supplemental Information for full details. In Figure 3, cells were fixed in 4% formaldehyde for 20 min and permeabilized for 5 min in PBS 0.2% Triton X-100. Coverslips were processed similarly to Toledo et al. (2013).

Statistics

Statistical significance was analyzed using Wilcoxon sum rank test, Student's t test, or two-way ANOVA, with Tukey's as appropriate, indicated in figure legends; further details are in the Supplemental Information. When appropriate, S phase cells were defined as the portion of cells where RPA2 > 10 a.u. (Figures 1B, 5E, and S2A), RPA2 > 20 a.u. (Figures 5D and S4C), or where RS occurs, increasing γH2AX intensity during HU treatment (Figure 1A).

Western Blot

Cell extracts were prepared in RIPA buffer, run on Novex 4%–12% Bis-Tris protein gels, probed for the antibodies indicated, and then secondary horseradish peroxidase (HRP) used (described fully in the Supplemental Information). Tubulin, GAPDH, and actin were loading controls; histone H3 was used for chromatin preparation loading controls.

Fiber Analysis

HEK293 T-Rex E2F6 or T98G were labeled with 25 μM CldU and then 250 μM IdU; see schematics. Fiber spreading and labeling were as in Petermann et al. (2010). Images were taken by confocal microscopy and analyzed with ImageJ. One hundred fifty to two hundred fibers were measured for each.

Chromatin Preparation

Cells were scraped from the dish in 400 μl buffer A and then Triton (0.1%) was added for 8 min on ice. The nuclear fraction was pelleted, washed with 200 μl buffer A, and resuspended in 400 μl buffer B for 30 min on ice. Chromatin fraction was pelleted and resuspended in 150 μl 15 mM Tris/0.5% SDS. The sample was spun before use. See the Supplemental Information for additional details.

FACS

Flow cytometry of DAPI/RPA2/γH2AX was performed as previously described (Forment et al., 2012). See the Supplemental Information for full details.

SUPPLEMENTAL INFORMATION

Supplemental Information includes Supplemental Experimental Procedures, five figures, and two movies and can be found with this article online at http://dx.doi.org/10.1016/j.celrep.2016.04.036.

AUTHOR CONTRIBUTIONS

C.B., A.E.H., B.R.P., and R.A.M.d.B. designed research. C.B., A.E.H., and B.R.P. performed research. C.B., A.E.H., and J.K.-V. analyzed data. C.B., A.E.H., and R.A.M.d.B. wrote the paper.

ACKNOWLEDGMENTS

We are grateful to Dr. R.M. Jones for kindly providing the DNA fiber protocol, Dr. J. Lukas for providing the RPA2-GFP plasmid, and Dr. J. Skotheim, Dr. M. Smolka, and Prof. B. Baum for critically reading the manuscript. We are particularly grateful to Prof. M. Marsh for his continual support. We thank the LMCB imaging facility for their help and all the people of the R.A.M.d.B. group for help and discussion. This work was supported by the MRC-UCL University Unit (Ref. MC_EX_G0800785) and funded by R.A.M.d.B.'s Cancer Research UK Programme Foundation Award.

Received: December 23, 2015
Revised: February 11, 2016
Accepted: April 5, 2016
Published: May 5, 2016

REFERENCES

Abraham, R.T. (2001). Cell cycle checkpoint signaling through the ATM and ATR kinases. Genes Dev. *15*, 2177–2196.

Bertoli, C., Klier, S., McGowan, C., Wittenberg, C., and de Bruin, R.A. (2013a). Chk1 inhibits E2F6 repressor function in response to replication stress to maintain cell-cycle transcription. Curr. Biol. *23*, 1629–1637.

Bertoli, C., Skotheim, J.M., and de Bruin, R.A. (2013b). Control of cell cycle transcription during G1 and S phases. Nat. Rev. Mol. Cell Biol. *14*, 518–528.

Branzei, D., and Foiani, M. (2010). Maintaining genome stability at the replication fork. Nat. Rev. Mol. Cell Biol. *11*, 208–219.

Buisson, R., Boisvert, J.L., Benes, C.H., and Zou, L. (2015). Distinct but concerted roles of ATR, DNA-PK, and Chk1 in countering replication stress during S phase. Mol. Cell *59*, 1011–1024.

Ciccia, A., and Elledge, S.J. (2010). The DNA damage response: making it safe to play with knives. Mol. Cell *40*, 179–204.

Cimprich, K.A., and Cortez, D. (2008). ATR: an essential regulator of genome integrity. Nat. Rev. Mol. Cell Biol. *9*, 616–627.

Forment, J.V., Forment, J.V., Walker, R.V., and Jackson, S.P. (2012). A high-throughput, flow cytometry-based method to quantify DNA-end resection in mammalian cells. Cytometry A *81*, 922–928.

Giangrande, P.H., Zhu, W., Schlisio, S., Sun, X., Mori, S., Gaubatz, S., and Nevins, J.R. (2004). A role for E2F6 in distinguishing G1/S- and G2/M-specific transcription. Genes Dev. *18*, 2941–2951.

Helmrich, A., Ballarino, M., and Tora, L. (2011). Collisions between replication and transcription complexes cause common fragile site instability at the longest human genes. Mol. Cell *44*, 966–977.

Hills, S.A., and Diffley, J.F. (2014). DNA replication and oncogene-induced replicative stress. Curr. Biol. *24*, R435–R444.

Huen, M.S., and Chen, J. (2008). The DNA damage response pathways: at the crossroad of protein modifications. Cell Res. *18*, 8–16.

Jackson, S.P., and Bartek, J. (2009). The DNA-damage response in human biology and disease. Nature *461*, 1071–1078.

Koundrioukoff, S., Carignon, S., Técher, H., Letessier, A., Brison, O., and Debatisse, M. (2013). Stepwise activation of the ATR signaling pathway upon increasing replication stress impacts fragile site integrity. PLoS Genet. *9*, e1003643.

Lossaint, G., Larroque, M., Ribeyre, C., Bec, N., Larroque, C., Décaillet, C., Gari, K., and Constantinou, A. (2013). FANCD2 binds MCM proteins and controls replisome function upon activation of s phase checkpoint signaling. Mol. Cell *51*, 678–690.

McGowan, C.H., and Russell, P. (2004). The DNA damage response: sensing and signaling. Curr. Opin. Cell Biol. *16*, 629–633.

Pellicioli, A., Lucca, C., Liberi, G., Marini, F., Lopes, M., Plevani, P., Romano, A., Di Fiore, P.P., and Foiani, M. (1999). Activation of Rad53 kinase in response to DNA damage and its effect in modulating phosphorylation of the lagging strand DNA polymerase. EMBO J. *18*, 6561–6572.

Petermann, E., and Helleday, T. (2010). Pathways of mammalian replication fork restart. Nat. Rev. Mol. Cell Biol. *11*, 683–687.

Petermann, E., Orta, M.L., Issaeva, N., Schultz, N., and Helleday, T. (2010). Hydroxyurea-stalled replication forks become progressively inactivated and require two different RAD51-mediated pathways for restart and repair. Mol. Cell *37*, 492–502.

Schlacher, K., Christ, N., Siaud, N., Egashira, A., Wu, H., and Jasin, M. (2011). Double-strand break repair-independent role for BRCA2 in blocking stalled replication fork degradation by MRE11. Cell *145*, 529–542.

Seiler, J.A., Conti, C., Syed, A., Aladjem, M.I., and Pommier, Y. (2007). The intra-S-phase checkpoint affects both DNA replication initiation and elongation: single-cell and -DNA fiber analyses. Mol. Cell. Biol. *27*, 5806–5818.

Tercero, J.A., Longhese, M.P., and Diffley, J.F. (2003). A central role for DNA replication forks in checkpoint activation and response. Mol. Cell *11*, 1323–1336.

Toledo, L.I., Altmeyer, M., Rask, M.B., Lukas, C., Larsen, D.H., Povlsen, L.K., Bekker-Jensen, S., Mailand, N., Bartek, J., and Lukas, J. (2013). ATR prohibits replication catastrophe by preventing global exhaustion of RPA. Cell *155*, 1088–1103.

Yamada, M., Watanabe, K., Mistrik, M., Vesela, E., Protivankova, I., Mailand, N., Lee, M., Masai, H., Lukas, J., and Bartek, J. (2013). ATR-Chk1-APC/CCdh1-dependent stabilization of Cdc7-ASK (Dbf4) kinase is required for DNA lesion bypass under replication stress. Genes Dev. *27*, 2459–2472.

Yamada, M., Masai, H., and Bartek, J. (2014). Regulation and roles of Cdc7 kinase under replication stress. Cell Cycle *13*, 1859–1866.

Zhang, Y.W., Otterness, D.M., Chiang, G.G., Xie, W., Liu, Y.C., Mercurio, F., and Abraham, R.T. (2005). Genotoxic stress targets human Chk1 for degradation by the ubiquitin-proteasome pathway. Mol. Cell *19*, 607–618.

Zou, Y., Liu, Y., Wu, X., and Shell, S.M. (2006). Functions of human replication protein A (RPA): from DNA replication to DNA damage and stress responses. J. Cell. Physiol. *208*, 267–273.

Cell Reports, Volume *15*

Supplemental Information

Sustained E2F-Dependent Transcription Is a Key Mechanism to Prevent Replication-Stress-Induced DNA Damage

Cosetta Bertoli, Anna E. Herlihy, Betheney R. Pennycook, Janos Kriston-Vizi, and Robertus A.M. de Bruin

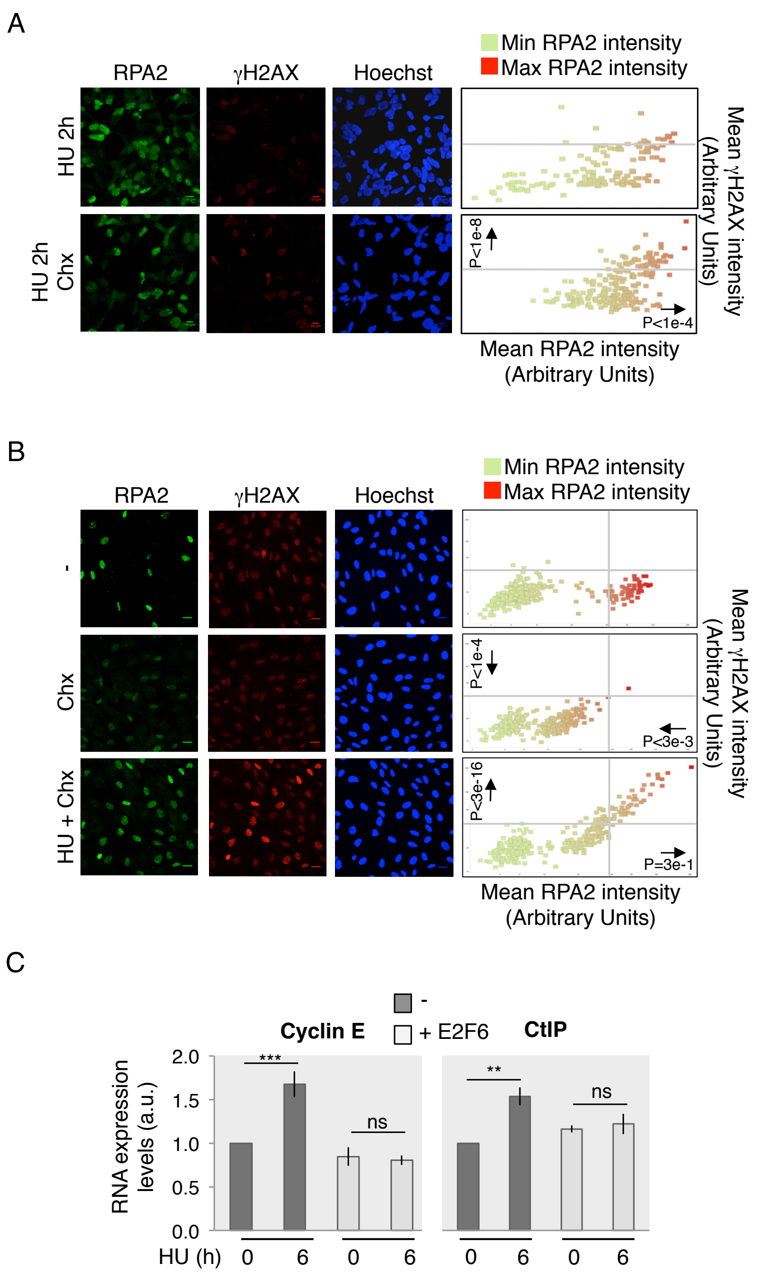

Figure S1, related to Figure 1. Active protein synthesis is required to prevent RS-induced DNA damage. A) Right, scatter plot of mean intensity of RPA2 (green, x-axis) against γH2AX (red, y-axis) of single nuclei, 2 hr HU -/+ Cycloheximide (Chx) in HEK293 T-Rex E2F6 cells. The logarithmic scale is identical for all. P values show significant differences on both axes with the Wilcoxon test. Arrows show increase/decrease of mean on each axis. Left, Representative immunofluorescence images showing chromatin-bound protein. Scale bar represents 10 µm. **B)** Right, scatter plot of mean intensity of RPA2 (green, x-axis) against γH2AX (red, y-axis) of single nuclei. Treatments shown, 7h, RPE1 cells. The logarithmic scale is identical for all. P values show significant differences (except for RPA2 between no treatment and HU + Chx) on both axes with the Wilcoxon test compared to control (-). Arrows show increase/decrease of mean on each axis. Left, Representative immunofluorescence images showing chromatin bound proteins. Scale bar represents 20 µm. **C)** RNA expression of HEK293 T-Rex E2F6 cells after HU treatment -/+ E2F6 overexpression (Doxy 2 µg/ml, 2 hour pretreat) for the time points shown. RNA was extracted and quantified by RT qPCR, normalised to GAPDH and normalised to HU time point 0. n=3, error bars show SEM. *** P<0.001, ** P<0.01 with ANOVA and Tukey's.

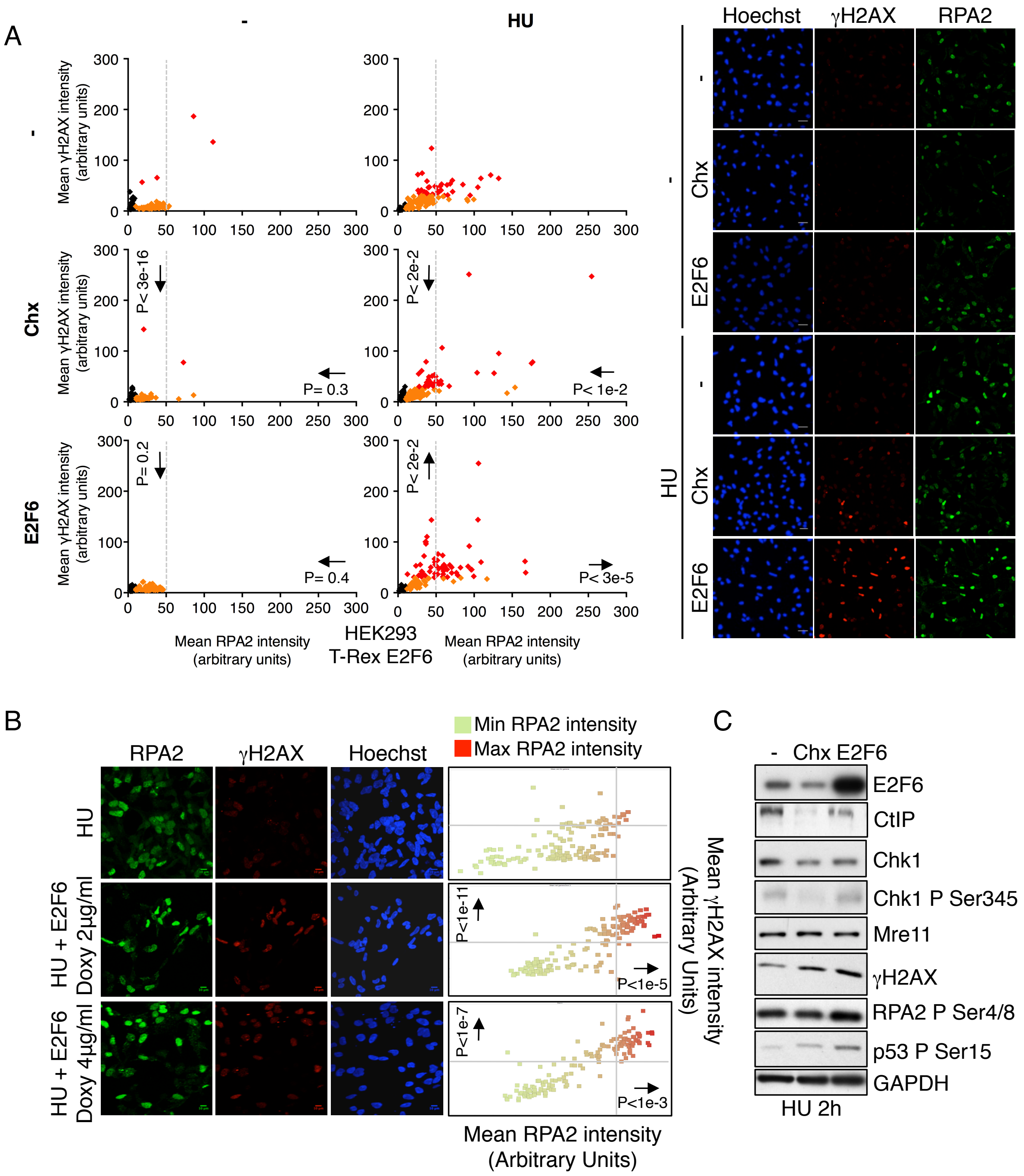

Figure S2, related to Figure 1. Active protein synthesis and E2F transcription is required to prevent RS-induced DNA damage. A) Left, scatter plot of mean intensity of RPA2 (green, x-axis) against γH2AX (red, y-axis) of single nuclei. Treatments shown, 2 hr HU, HEK293 T-Rex E2F6. Black dots show non S phase cells (RPA2<10 a.u.), orange and red dots show low and high levels of γH2AX respectively (arbitrary threshold γH2AX=30 a.u.). P values show differences of S phase cells on both axes with the Wilcoxon test compared to control (- or HU treated as appropriate). Arrows show increase/decrease of mean on each axis. Right, representative images. Scale bar represents 20 µm. **B)** Right, scatter plot of mean intensity of RPA2 (green, x-axis) against γH2AX (red, y-axis) of single nuclei. Treatments shown, 2 hr HU, HEK 293 T-Rex E2F6 cells. Doxy 1.5 hr pretreat. The logarithmic scale is identical for all. P values show significant differences on both axes with the Wilcoxon test compared to control (HU). Arrows show increase/decrease of mean on each axis. Left, Representative immunofluorescence images showing chromatin bound proteins. Scale bar represents 10 µm. **C)** Western blot of WCE, HEK293 T-Rex E2F6 cells, treatments shown, 2h HU. E2F6 (Doxy, 2 µg/ml, 2 hr pretreat).

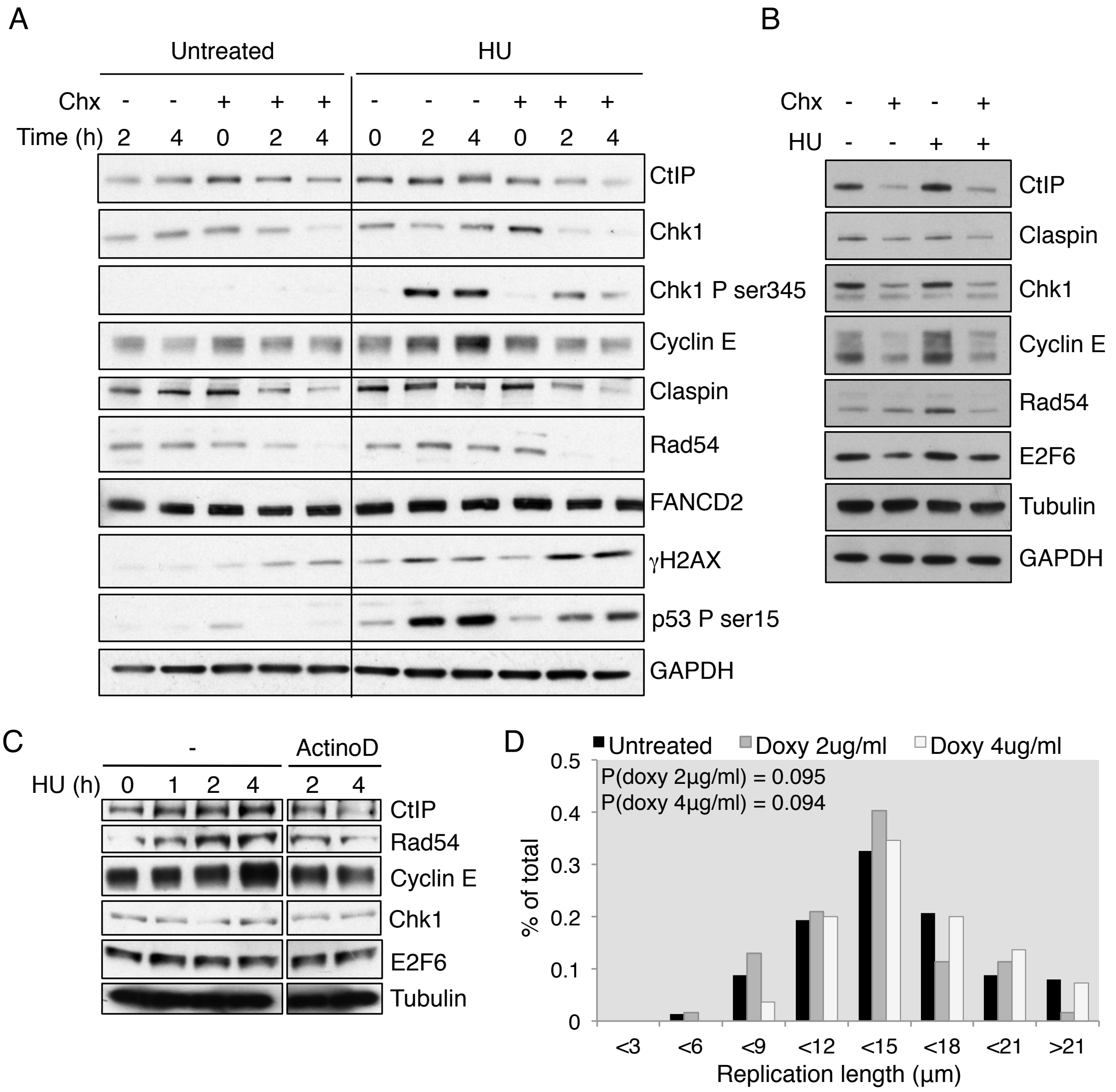

Figure S3, related to Figure 2 and 3. Protein synthesis and E2F transcription are required to maintain the levels of checkpoint proteins during RS. A) Western blot of WCE, T98G cells, treatments and times shown. **B)** Western blot of WCE, RPE1 cells, treatments shown, 4 h. **C)** Western blot of WCE, HEK293 T-Rex E2F6 cells, treatments and times shown. ActinoD is transcriptional inhibitor Actinomycin D. **D)** DNA fiber analysis in HEK293 T-Rex E2F6 cells. Bar graphs of green track lengths. Cells were treated for 3.5 hr -/+ Doxycycline at 2 μg/ml or 4 μg/ml as indicated (timing equivalent to 1.5 hr pretreat then 2 hr HU treatment with Doxy in Figure 3A). Cells were pulse labelled with CldU (red) for 20 min, then IdU (green) for 20 min and the lengths of green tracks measured. P values show no significant difference in track length with E2F6 overexpression with the Student's T test.

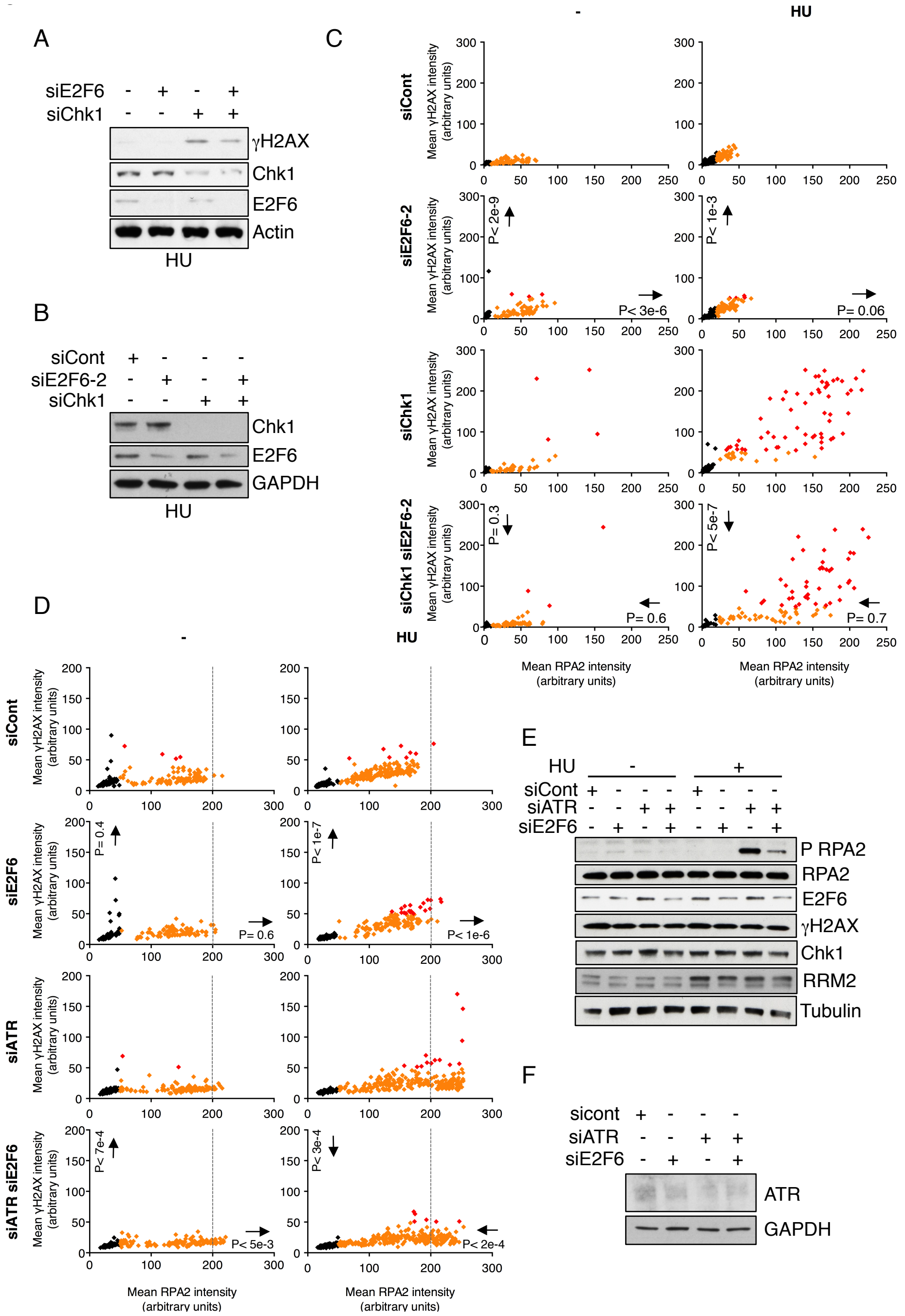

Figure S4, related to Figure 4. E2F activity is required and sufficient to reduce RS-induced DNA damage in a compromised checkpoint response. A) Western blot WCE of serum starvation-synchronized T98G cells transfected with siRNA shown, and released in complete medium with HU for 30 hours. **B)** Western blot of WCE, RPE1 cells, transfected with the reported siRNA, 4h HU. **C)** Scatter plot of mean intensity of RPA2 (x-axis) against γH2AX (y-axis) of single nuclei transfected with the reported siRNA -/+ 4 hr HU, RPE1 cells. Black dots show non S phase cells (RPA2<20 a.u.), orange and red dots show low and high levels of γH2AX respectively (arbitrary threshold γH2AX=50 a.u.). P values show differences on both axes of S phase cells (RPA2 > 20 a.u) with the Wilcoxon test compared to the non-siE2F6 equivalent. Arrows show increase/decrease of mean. **D)** Scatter plot of mean intensity of RPA2 (x-axis) against γH2AX (y-axis) of single nuclei transfected with the reported siRNA, -/+ 4 hr HU, RPE1 cells. Black dots show non S phase cells (RPA2<50 a.u.), orange and red dots show low and high levels of γH2AX respectively (arbitrary threshold γH2AX=50 a.u.). P values show differences on both axes with the Wilcoxon test compared to the non siE2F6 equivalent. Arrows show increase/decrease of mean. **E)** Western blot of WCE, RPE1 cells transfected with the reported siRNA -/+ 4 hr HU. **F)** Western blot of WCE, RPE1 cells transfected with the reported siRNA.

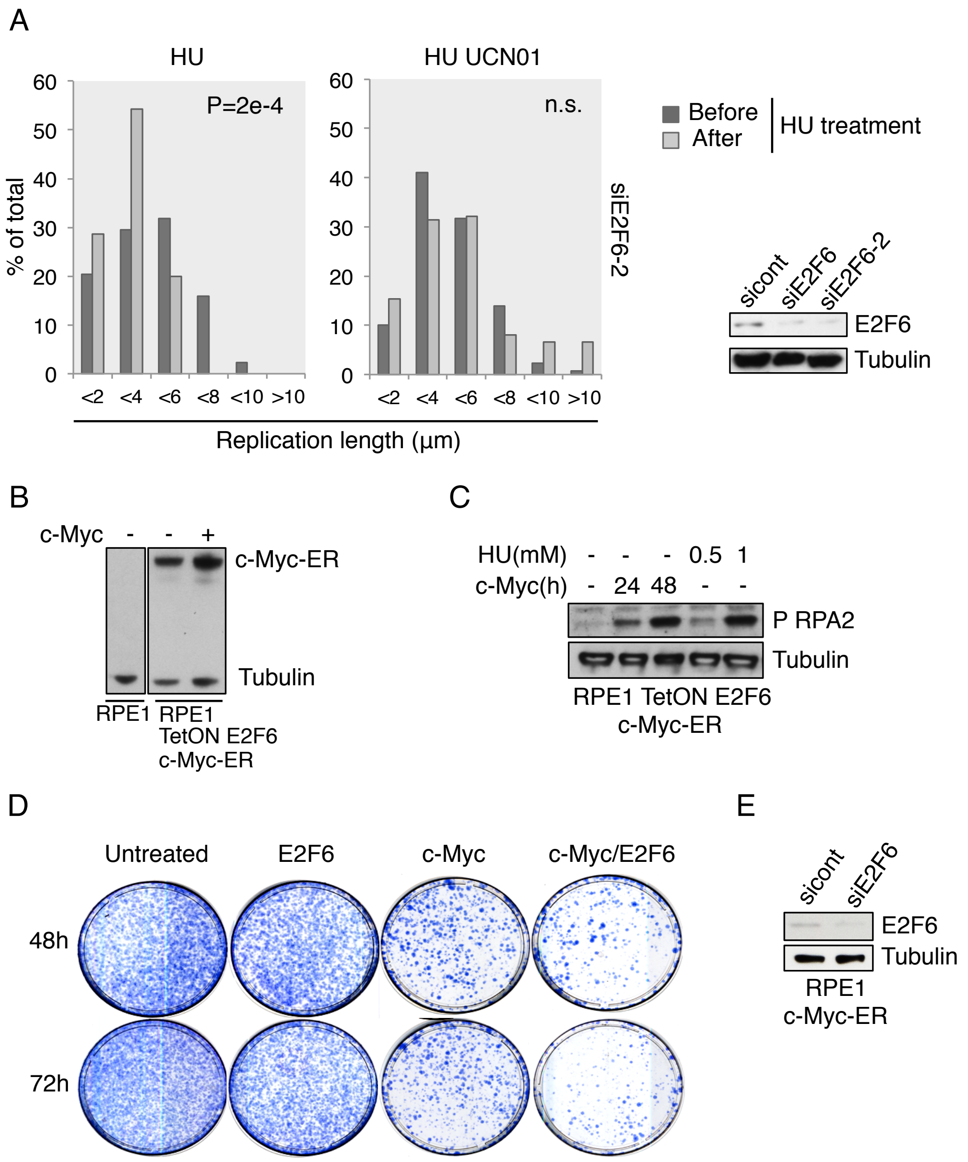

Figure S5, related to Figure 4 and Figure 5. E2F activity is required for tolerance to oncogene-induced RS. **A)** Experimental design as in Figure 4E, T98G cells. Left, bar graphs of length of DNA tracks before and after HU treatment with a second siRNA targeting E2F6 (E2F6-2) -/+ UCN01. P values show difference with the Wilcoxon test. Right, western blot showing the silencing of E2F6 with the two siRNA compared to control siRNA. **B)** Western blot showing the expression of c-Myc-ER in RPE1 TetON E2F6 c-Myc-ER cells compared to non-infected RPE1. Tubulin is the loading control. **C)** Western blot of WCE, RPE1 TetON E2F6 c-Myc-ER cells treated with different RS-causing agents – c-Myc overexpression (times shown) or HU 7 hr (concentrations shown). **D)** Colony formation assays showing the survival and proliferation of RPE1 TetON E2F6 c-Myc-ER cells exposed to c-Myc-ER induction (4OH-T, 100nM), E2F6 overexpression (Doxy 2 µg/ml), or a combination as shown, for the reported time, then diluted in normal medium and allowed to grow for 10 days before being fixed and stained. **E)** Western blot of WCE, RPE1 TetON E2F6 c-Myc-ER treated with the shown siRNA, samples from Figure 5E.

Supplemental Experimental Procedures

Cell culture

HEK293 T-Rex E2F6 and T98G cells were maintained in DMEM (Gibco, 41965) 10% FBS (Sigma, F7524) supplemented with Pennicillin/Streptomycin (Gibco, 15140). RPE-1 hTERT and RPE1 TetON E2F6 were maintained in DMEM or DMEM/F12 (Gibco, 31331) supplemented with Sodium Carbonate (Gibco, 25080) and Pennicillin/Streptomycin, as recommended by ATCC. HEK293 T-Rex E2F6 cells were previously described (Bertoli et al., 2013) and were maintained in 5 μg/ml Blasticidin and 100 μg/ml Zeocin. RPE1 TetON E2F6 were created for this study. Cells were transfected with Lipofectamine 2000 (Invitrogen) following manufacturers instructions using the plasmid pcDNA4/TO E2F6. Transformed cells were selected in 5 μg/ml Blasticidin and 200 μg/ml Zeocin and colonies tested for Doxycycline dependent E2F6 expression. RPE1 TetON E2F6 were maintained in 5 μg/ml Blasticidin and 100 μg/ml Zeocin. RPE1 TetON E2F6 c-Myc-ER was created for this study. Cells were obtained by retroviral infection of RPE1 TetON E2F6 using the pBABE c-Myc-ER plasmid (Addgene plasmid 19128) (Ricci et al., 2004). Infected cells were then selected in puromycin 5 μg/ml and the surviving cells used for the assays. The RPE1 TetON E2F6 c-MycER cells were maintained in phenol-free DMEM/F12 10% charcoal-treated bovine serum with the supplements described above and in 5 μg/ml Blasticidin, 100 μg/ml Zeocin and 2 μg/ml Puromycin.

siRNA Transfection

The sequences used were: siCont (LacZ) AACGUACGCGGAAUACUUCGA, siE2F6 AAGGAUUGUGCUCAGCAGCUG (**Figures 4ABCDF, 5E, S4ADE, S5E**), siE2F6-2 AAACAAGGUUGCAACGAAAUU (**Figures S4B, S4C, S5A**), siChk1 GAAGCAGUCGCAGUGAAGAUU (Bertoli et al., 2013) (**Figures 4D, S4A**), SMARTpool: siGENOME CHEK1 siRNA (M-003255-04-005, Dharmacon GE Life Sciences) (**Figures 4ABC, S4B,S4C**), siATR UUGUAGAAAUGGAUACUGA (validated Silencer Select s536, Thermo Fisher Scientific).

Immunofluorescence

Figures 1A, B, 4B, C, 5D, E, and Figures S1A, B, S2A, B, S4C,D : Cells were pre-extracted for 1 min in ice cold PBS 0.2% Triton-X100 (or 0.15% for HEK293 T-Rex cells), then fixed with 4% formaldehyde for 20 min and processed similarly to (Toledo et al., 2013). Briefly, coverslips were blocked in 1% BSA for 1 hr and incubated in primary antibodies overnight at 4˚C – RPA32 (RPA2) (1:500, Ms, MABE285, Millipore) and Phospho-Histone H2A.X (γH2AX) (Ser139) (1:400, Rb, 20E3, Cell Signaling Technology). Coverslips were incubated in secondary antibodies for 1 hour at RT – anti-mouse Alexa Fluor 488, and anti-rabbit Alexa Fluor 647 both 1:2000 (LifeTechnologies), incubated 5 minutes in Hoechst 1:10,000, (Invitrogen), and mounted with Fluoroshield (Sigma). Images were obtained with a Leica TCS SP5, or a Leica TCS SPE2, 63x objective lens. Images were processed in Fiji – the Hoechst channel was used to segment nuclei with a median filter and Otsu thresholding. The mean gray value of each channel was then measured for each nucleus. The signals for individual cells were then plotted using Spotfire or Prism software.

Figure 3C,D: Cells were fixed with 4% formaldehyde for 20 min and permeabilised for 5 min in PBS 0.2% Triton-X100. Coverslips were then processed as above with RPA32 (RPA2) antibody (1:1000, Ms, MABE285, Millipore). The percentage of cells containing RPA2 foci was manually counted in Fiji.

Statistics

Figure 1A,B, 5D,E and Figure S2A, S4C: Statistical significance was analyzed using the Wilcoxon sum rank test; two sided, using the R function Wilcox.test. Only S phase cells were analyzed, defined as the portion of cells where RPA2 > 10 a.u. (Figure 1B, 5E, S2A), RPA2 > 20 a.u. (Figure 5D, S4C), or where RS occurs, as seen by an increase in γH2AX intensity during HU treatment (Figure 1A). **Figures 4B and Figures S1A,B, S2B, S4D:** Statistical significance was analyzed for both RPA2 and γH2AX against control conditions using the Wilcoxon sum rank test, two sided, using the R function Wilcox.test. **Figures 1C, 3A, B and Figure S3D:** Statistical significance was analyzed by Student's T test. **Figure 3C:** Statistical significance was analyzed by two-way ANOVA (using the aov() function of R), the multiple comparisons of the means was performed by Tukey's honest significant differences, using the R function TukeyHSD().*** P<0.001, * P<0.05. **Figure 4F and Figure**

S5A: Statistical significance was analyzed for differences between mean track length after – mean track length before HU treatment using the Wilcoxon sum rank test, two sided, using the R function Wilcox.test.
Figure S1C: Statistical significance was analyzed by two-way ANOVA, the multiple comparisons of the means was performed by Tukey's honest significant differences, using Prism software. *** $P<0.001$, ** $P<0.01$.

Western blot

Cell extracts were prepared in RIPA buffer (Tris-HCl pH7.5 20mM, NaCl 150mM, EDTA 1mM, EGTA 1mM, NP40 1%, NaDoc 1%) containing phosphatase inhibitor cocktails 2 and 3, 1:1000 (Sigma P5726 and P0044) and protease inhibitor cocktail, 1:1000 (Sigma, P8340). Run on Novex 4-12% Bis-Tris protein gels. The following antibodies were used for Western blot analysis, all were used at 1:1000 unless otherwise stated: Claspin (Rb, ab3720), CtIP (Rb, ab70163), Mre11 (Ms, ab214), Rad54 (Ms, ab11055), Histone H3 (Rb, ab1791) from Abcam, Chk1 (Ms, DCS-310, sc-56291), Cyclin E (HE12) (Ms, sc-247), Rad51 (Rb, H-92, sc-8349), Cdc7 (1:200, Ms, DCS-341, sc-56274), PCNA (1:2000, Ms, F-2, sc-25280), ATR (Gt, N-19, sc-1887), Myc 9E10 (sc-40) from Santa Cruz, GAPDH (Ms, GT239) from GeneTex, Tubulin (1:2000, Ms, MAB3408), RPA34-20 (Ms, MABE285), E2F6 (Ms, MABE57) from Millipore, FANCD2 (1:2000, Rb, NB100-182) from Novus biological, RPA32 (RPA2) (S4/S8) (Rb, A300-245A-3) from Bethyl laboratories, Phospho-Histone H2A.X (γH2AX) (Ser139) (Rb, 20E3), Phospho-Chk1 (ser345) (Rb, 2341), p53 (Ser15) (Rb) from Cell Signaling Technology, Actin (Rb, A2066) from Sigma, RRM2 (Ms, M01, 1E1), E2F6 (Ms) a kind gift of Dr Jacky Lees. Secondary antibodies used were Goat anti-mouse and anti-rabbit HRP conjugated (Fisher Scientific, PA1-74421 Mouse, PI-31460 Rabbit).

Fiber analysis

HEK-293 T-Rex E2F6 or T98G were labeled with 25 µM CldU followed by 250 µM IdU, as in the schematics. Fiber spreading and labeling was performed as in (Petermann et al., 2010). Images were taken by confocal microscopy and analyzed with ImageJ. 150-200 fibers were measured for each experiment.

RNA expression

RNA was extracted with the Qiagen RNeasy Mini kit following manufacturers protocol. RT qPCR SYBR assay was carried out with Mesa Blue following manufacturers instructions, 80 ng RNA per well, experimental triplicates, BioRad CTX Connect qPCR machine. RNA levels were normalized to GAPDH. Experiment was done in biological triplicate. Primers used were: GAPDH Fw 5'- GAAATCCCATCACCATCTTCCAGG-3', Rv 5'-GAGCCCCAGCCTTCTCCATG-3', Cyclin E Fw 5'-TGTCCTGGATGTTGACTGCCTTGA-3', Rv 5'-TGTCGCACCACTGATACCCTGAAA-3', CtIP, Fw 5'- CTGCTTGGGCACACGTGTAAG-3', Rv 5'-TGGAATGTAGCGGAATCGGTGTCT-3'.

Chromatin Preparation

Buffer A (Hepes pH 7.9 10 mM; KCl 10 mM; MgCl 1.5 mM; Sucrose 0.34 M; Glycerol 10 %; DTT 1 mM; and protease and phosphatase inhibitors as recommended by the supplier). Buffer B (3 mM EDTA; 0.2 mM EGTA; protease and phosphatase inhibitors). The nuclear fraction was pelleted at 1300 g, 5 min, 4˚C. The chromatin fraction was pelleted at 1700 g, 5 min, 4˚C. The sample was spun at 9600 g, 5 min, 4˚C before use.

FACS

Flow cytometry was performed as described previously for DAPI/RPA2/γH2AX in (Forment et al., 2012). Phospho-Histone H2A.X (γH2AX) (Ser139) (Rb, 20E3) from Cell Signaling Technology used 1:200 and RPA2 (Ms, 9H8, ab2175) from Abcam used 1:250. Samples were measured on a BD LSRII flow cytometer using DIVA software (BD) and analyzed using FlowJo software.

Survival Assay

RPE1 TetON E2F6 c-MycER were treated as shown in Figure S5D for 48 or 72 hours, they were then diluted and left to grow in 10cm dishes until colonies were visible. Cells were fixed and stained as in Bertoli et al, 2013.

Supplemental References

Bertoli, C., Klier, S., McGowan, C., Wittenberg, C., and de Bruin, R.A. (2013). Chk1 Inhibits E2F6 Repressor Function in Response to Replication Stress to Maintain Cell-Cycle Transcription. Curr Biol.

Forment, J.V., Walker, R.V., and Jackson, S.P. (2012). A high-throughput, flow cytometry-based method to quantify DNA-end resection in mammalian cells. Cytometry Part A : the journal of the International Society for Analytical Cytology *81*, 922-928.

Petermann, E., Orta, M.L., Issaeva, N., Schultz, N., and Helleday, T. (2010). Hydroxyurea-stalled replication forks become progressively inactivated and require two different RAD51-mediated pathways for restart and repair. Mol Cell *37*, 492-502.

Ricci, M.S., Jin, Z., Dews, M., Yu, D., Thomas-Tikhonenko, A., Dicker, D.T., and El-Deiry, W.S. (2004). Direct repression of FLIP expression by c-myc is a major determinant of TRAIL sensitivity. Mol Cell Biol *24*, 8541-8555.

Toledo, L.I., Altmeyer, M., Rask, M.B., Lukas, C., Larsen, D.H., Povlsen, L.K., Bekker-Jensen, S., Mailand, N., Bartek, J., and Lukas, J. (2013). ATR prohibits replication catastrophe by preventing global exhaustion of RPA. Cell *155*, 1088-1103.
